# Quantum Single-Path Transmission Optimization of Complex Networks

**DOI:** 10.3390/e28080900

**Published:** 2026-08-10

**Authors:** Zhengyi Wang, Feng Gao, Yunqing Xu, Xiaohui Wang, Jingyang Fang

**Affiliations:** 1 School of Control Science and Engineering, Shandong University, Jinan 250061, China; 202120614@mail.sdu.edu.cn (Z.W.); 202314817@mail.sdu.edu.cn (Y.X.); jingyangfang@sdu.edu.cn (J.F.); 2 School of Electrical Engineering, Shandong University, Jinan 250061, China; wangxiaohui@sdu.edu.cn

**Keywords:** single-path flow optimization, quantum optimization, QUBO model, cost-flow bias coefficient, spline interpolation function

## Abstract

Single-path transmission optimization is a core task for resource scheduling and operation of complex networks, which requires coordinated optimization of transmission cost and flow. Classical algorithms bear heavy computational loads in high-dimensional decision spaces as networks grow. This paper constructs a hybrid quantum model integrating quantum approximate optimization algorithm (QAOA) and cubic spline interpolation. Paths, discrete flows, and trade-off coefficients are unified within a quadratic unconstrained binary optimization (QUBO) model. Least-squares fitting converts native parameters into QUBO coefficients, whose fitting errors are measured to verify robustness and penalty sensitivity, and auxiliary variables eliminate high-order terms to exponentially cut qubit consumption. QAOA narrows the feasible range via global coarse search, and cubic spline interpolation further yields precise continuous flow values. Powered by quantum superposition for parallel full-space exploration, the framework avoids repeated modeling for separate bias coefficients. Mixed integer programming (MIP) and genetic algorithm (GA) are adopted as comparative benchmarks. For the small-scale network instance, the relative error between the proposed method and the global optimum solved by MIP is less than 1%. For the large-scale case, the overall error of our approach remains within an acceptable range even when discrepancies exist between results yielded by classical algorithms.

## 1. Introduction

Single-path flow transmission constitutes a critical issue in flow scheduling and routing optimization for complex networks. A complex network refers to a network structure composed of a large number of nodes and complex connections between these nodes [1,2]. Its research scenario is usually modeled by applying appropriate optimization algorithms on a directed weighted graph [3], where nodes represent functional units and edges represent directional connections weighted with cost or flow attributes [4]. The core lies in selecting a cost-effective path between source and sink node pairs for flow transmission, with the flow rate optimized within the path capacity limit [5]. This problem widely exists in many critical engineering applications, such as power system flow analysis [6,7], data center network flow optimization [8], communication network parameter optimization [9,10,11], traffic route planning, and supply chain logistics routing [12,13,14].

However, the problem of optimizing single-path traffic transmission has a relatively high computational complexity. On the one hand, path flow and unit cost are mutually coupled. In different scenarios, cost and flow must be traded off via bias coefficient [15,16], generating a parameter-driven combinatorial optimization problem [17,18]. On the other hand, as network scale and topological complexity increase, the number of feasible paths grows rapidly. Moreover, unit flow cost often exhibits non-linear variations with network load due to time delay in transportation networks [19], uncertainty of transmission loss in power systems [20], and congestion cost in communication networks [21], making collaborative optimum between flow and cost hard to achieve.

Traditional optimization algorithms can be divided into exact methods that guarantee finding the global optimal solution and approximation methods. Exact methods include the Dijkstra algorithm [22], Floyd–Warshall algorithm [23], branch-and-bound algorithm [24], mixed-integer programming [25,26], etc. However, constrained by the NP-hard nature, they are only applicable to small and medium-scale networks and are prone to computational bottlenecks due to the combinatorial explosion of decision variables in large-scale networks [27]. Approximation methods, such as heuristic algorithms like genetic algorithms and ant colony optimization [28,29], can solve problems in polynomial time but cannot guarantee the global optimal solution [30].

To address the aforementioned issues, we propose a quantum optimization scheme for single-path transmission. Quantum optimization problems can be modeled as quadratic unconstrained binary optimization (QUBO) problems, mapped to an Ising Hamiltonian [31,32], and then solved by algorithms such as the quantum approximate optimization algorithm (QAOA) or quantum annealing (QA) [33,34,35,36]. Currently, quantum optimization algorithms have been widely applied to complex network-related optimization problems, including the maximum cut problem [37,38,39], graph coloring problem [40,41], and route planning [42,43,44,45,46]. By leveraging quantum coherence to reduce redundant computations, these algorithms significantly improve solution quality and can easily find optimal solutions for small-scale graphs. However, when adapting quantum optimization models to multi-scale single-path optimization scenarios, existing schemes have obvious limitations: the number of path variables is equal to the number of qubits, leading to a linear increase in qubit demand as topological complexity surges, resulting in severe resource bottlenecks [47,48]. On the other hand, multiple optimization parameters make it difficult to satisfy the QUBO condition. Therefore, there is an urgent need for innovations at the algorithmic level to unlock the theoretical advantages of quantum hardware.

In this paper, logarithmic-scale qubit encoding is applied to candidate paths to prevent decision variable count from linearly increasing with network scale. The maximum single-path flow is discretized into levels, enabling a unified description of flow and path variables within the same binary variable combinatorial framework. Additional qubits encode the cost-flow bias coefficient, allowing different coefficients to be integrated into a single quantum model. Unlike traditional algorithms that require sequential optimization under each coefficient, the proposed model directly yields optimal values for all coefficients simultaneously. The QAOA is used to solve the model and find the minimum-cost path for each bias coefficient. Notably, finer discretization of the bias coefficient and flow level improves result precision but requires more qubits; alternatively, a continuous interpolation function can smooth the cost-flow relationship to obtain accurate optimal solutions, which is adopted in this paper. Thus, the proposed quantum optimization scheme achieves single-path transmission optimization under various bias coefficients in polynomial time.

The rest of this paper is organized as follows: Section 2 first introduces the optimization model for single-path flow transmission and analyzes the combinatorial optimization characteristics of the cost-flow bias coefficient. It then proposes a novel quantum modeling approach consisting of logarithmic quantum path encoding, discretized flow variables, and bias parameter embedding. Afterwards, the complete global optimization workflow for solving the quantum model via the QAOA algorithm is elaborated in detail, and cubic spline interpolation is introduced to refine continuous flow values for the discrete optimal paths obtained by quantum computation. Section 3 verifies the performance of the proposed method through numerical experiments. Finally, Section 4 draws the conclusion and discusses the future work.

## 2. Materials and Methods

### 2.1. Quantum Model of Single-Path Flow Transmission

The complex network referred to in this paper is a directed weighted graph featuring small-world properties, non-uniform node degree distribution, multiple feasible transmission paths between source and sink nodes, and a non-linear coupling relationship between edge transmission cost and traffic load, which is different from simple lattice networks or fully connected networks. Also known as the quantum Internet, a quantum network relies on quantum communication technologies to generate, transmit, and utilize quantum state resources, enabling interconnection between quantum information processing systems or nodes such as quantum computers and quantum sensors as well as the transmission of quantum state information to further boost the capacity of quantum information transmission and processing [49]. The classical complex network discussed in this paper transmits classical bit information via optical and electrical signals, with data flows as the core transmission carrier. In contrast, quantum networks take qubits and quantum entanglement as core carriers to realize the transmission of quantum state information, including quantum key distribution and quantum teleportation. Both classical complex networks and quantum networks can be mathematically abstracted into a graph model *G*(*V*,*E*), where nodes stand for functional units and edges represent directional transmission channels.

**Figure 1:**
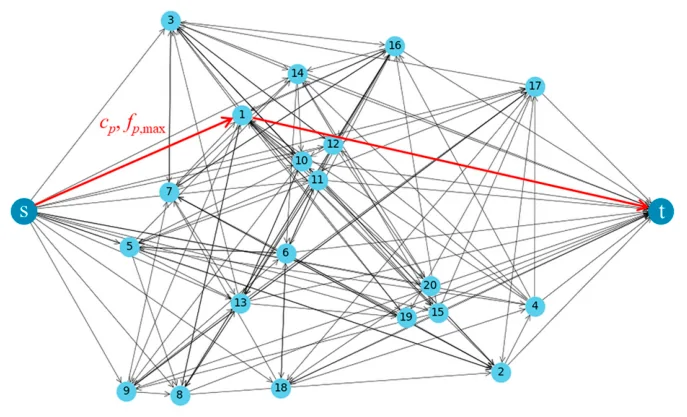
Schematic diagram of flow transmission in a directed network. The numbers inside the circles represent node indices, and the arrows indicate the propagation direction of flow. Darker colors and larger fonts mark the source s and sink t. The red path indicates a transmission path from s to t. Each path *p* contains several edges, each path has its unit cost *c_p_* and maximum flow *f_p_*_,max_.

Given a directed complex network *G*(*V*,*E*), where *V* is the set of nodes and *E* is the set of directed edges, each edge (*m*,*n*) ∈ *E* has a maximum capacity *u_mn_* and a unit flow cost *c_mn_*. After designating a source node s and a sink node t, one path should be selected from the *p* feasible paths between s and t as illustrated in Figure 1 for flow transmission under the constraints of capacity and flow conservation, such that the total cost from s to t is minimized.

During transmission, the maximum flow capacity *f_p_*_,max_ of a single path is determined by the edge with the minimum capacity in the path, which is known as the “bucket effect” [50,51,52,53]. In general, *f_p_*_,max_ is positively correlated with *c_p_*. In addition, the unit flow cost exhibits a non-linear relationship with the flow: the cost rises rapidly under high load due to increased loss and also deviates from linearity under low load due to the unavoidable inherent consumption, which means purely pursuing large flow or limiting the flow to a low level, which can both result in high total cost. Such a non-linear relationship is a challenge for single-path transmission optimization.

Before optimization, we first need to search out all paths from the specified source node s to the sink node t, including all nodes or edges traversed. Generally, for a given network, the paths between any node can be listed offline. Here, we recommend the depth-first search (DFS) algorithm to search all path combinations, whose pseudocode is given in Appendix A.

Subsequently, we first construct a conventional optimization model for single-path transmission, which corresponds to a bi-objective optimization problem. This optimization task involves two conflicting objectives, namely the cost function *C*(*p*) and the flow function *F*(*p*), and the two objectives cannot reach their respective optimal values simultaneously. The complete mathematical formulation of the bi-objective optimization model is presented in Equation (1):

(1) $$\left\{\begin{array}{l} \underset{p}{\min}\;\;\;\;C(p)=\sum_{(m,n)\in E_{p}}c_{mn}\cdot f_{p}\\ \underset{p}{\max}\;\;\;\;F(p)\\ s.t.\;\;\;\;0\leq F(p)\leq f_{p,\max},\;\;\;\;f_{p,\max}=\underset{(m,n)\in E_{p}}{\min}\;\;\;\;u_{mn} \end{array}\right.$$

here, *C*(*p*) denotes the total transmission cost of path *p*, *F*(*p*) stands for the flow of the candidate path, and the feasible region is constrained by the path capacity derived from the bucket effect. *f_p_* refers to the flow value of the *p*-th path. The total cost of a single path is calculated by summing the flow cost *c_mn_* of all edges contained in this path, and there exists a non-linear relationship between cost and flow. Their respective optimal solutions are generally contradictory, which indicates that we cannot simultaneously achieve the minimum cost and maximum flow. Accordingly, a cost-flow bias coefficient *λ* ∈ [0, 1] is introduced to convert the two conflicting objectives into a unified single-objective function, and the scalarized optimization objective is formulated as Equation (2):

(2) $$\underset{p}{\min}\;\;H(p,f_{p})=\lambda \cdot C(p)-(1-\lambda )\cdot F(p)$$

When approaching 1, the optimization objective tends to pure cost minimization, when *λ* approaches 0, it tends to pure flow maximization.

Next, we elaborate on the selection and calibration methods of the cost-flow bias coefficient *λ*. As the core parameter for single-path transmission optimization, *λ* governs the trade-off tendency between transmission cost and flow volume, and its value is determined by practical scheduling requirements. For cost-sensitive networks such as long-distance backbone optical communication networks, regional power transmission grids with substantial power transmission loss charges, and enterprise private data centers, *λ* should be set close to 1 to prioritize cost minimization at the expense of moderate throughput reduction. For throughput-sensitive networks, including emergency communication for disaster rescue, industrial real-time control systems, and urban congested traffic networks, *λ* ought to approach 0 to maximize flow volume regardless of moderate increases in unit transmission cost. To conduct comprehensive bi-objective analysis, the continuous feasible interval [*λ*_min_, *λ*_max_] of *λ* is uniformly discretized into multiple candidate values to systematically traverse all trade-off combinations. Solving the optimization model corresponding to each discrete *λ* yields a full set of feasible optimal solutions covering all cost-flow trade-off states. Decision-makers can complete the calibration of *λ* by filtering these solutions according to customized engineering indicators, such as the maximum allowable cost threshold and the minimum required transmission flow.

To render the problem tractable, the normalization of cost and flow is necessary since they differ greatly in dimension and magnitude. Let the original ranges of cost and flow be [*c*_min_, *c*_max_] and [*f*_min_, *f*_max_], respectively, and their normalized forms are defined as:

(3) $$\begin{array}{l} c^{*}=\frac{c-c_{\min}}{c_{\max}-c_{\min}}\\ f^{*}=\frac{f-f_{\min}}{f_{\max}-f_{\min}} \end{array}$$

where c* and f* ∈ [0, 1] represent the normalized dimensionless cost and flow variables, respectively.

In real networks, there exists a fixed total flow demand *F* between the source and the sink, which serves as an external constraint for the optimization problem. In this scenario, the optimization model defined by Equations (1) and (2) enables the two conflicting optimization objectives of minimum cost and maximum flow to achieve optimal values under different bias coefficients, thereby realizing the global minimum transmission cost under a fixed total flow demand. If *F* does not exceed the path capacity, it can be directly used as the upper bound constraint on the flow. If *F* exceeds the single-transmission capacity, the total transmission can be completed in multiple times while keeping the path and its corresponding flow unchanged.

Next, we will build the corresponding quantum optimization model for single-path transmission. There could be a lot of candidate paths searched out offline. We employ the amplitude encoding to represent the candidate paths. The advantage is that only a few qubits can represent all candidate paths. Specifically, the indexes of total *P* paths can be encoded using *K* = ⌈log_2_*P*⌉ binary bits, and paths are represented by combinations of binary variables *r_k_* ∈ {0, 1}, as shown in the following equation:

(4) $$p=\sum_{k=0}^{K}2^{k}r_{k}$$

thus, the path selection is determined by the combination of *K* binary bits.

The continuous flow variable is discretized with the maximum flow of the path as the upper bound. The flow range of each path is divided into *L* discrete levels, and binary variables *d_i_* ∈ {0, 1} are introduced to represent the flow level, where the total number of variables is *I* = ⌈log_2_*L*⌉. The path flow can be expressed as:

(5) $$f_{p}=\sum_{i=0}^{I}2^{i}\cdot d_{i}\cdot \frac{f_{p,\max}}{L-1}$$

thus, the maximum flow *f_p_*_,max_ is divided into *L* − 1 uniformly discrete values.

To adopt quantum optimization methods and demonstrate the logarithmic advantage of quantum encoding, the optimization models in Equations (1) and (2) are converted into the QUBO form. The combined binary variable vector is **x** = [*r*_1_,…, *r*_k_, *d*_1_,…, *d_i_*], the cost and flow functions are denoted as *C*(**x**) and *F*(**x**), respectively. The QUBO model expressions for cost and flow are given as follows:

(6) $$\begin{array}{l} C(x)=x^{T}Q_{C}x+q_{C}^{T}x+c_{C}\\ F(x)=x^{T}Q_{F}x+q_{F}^{T}x+c_{F} \end{array}$$

where **Q_C_** and **Q_F_** are symmetric matrices representing the quadratic coefficient vectors for cost and flow, respectively. **q_C_** and **q_F_** are the linear coefficient vectors, and **c_C_** and **c_F_** are the constant term vectors.

In Equation (4), we use a combination of path-representing binary variables **r** = [*r*_1_, *r*_2_,…, *r_k_*] to denote the binary number of path indices. In Equation (5), a combination of flow binary variables **d** = [*d*_1_, *d*_2_, …, *d_i_*] is used to represent discrete flow values, reducing the number of decision variables to a logarithmic scale compared to the number of paths. The linear or cross terms of **x** along with their weighted sum correspond to the discrete flow values or cost values of the respective paths, which need to be multiplied by QUBO coefficients and weighted summed before the linear and quadratic terms. To this end, coefficient fitting is employed to map objectives and constraints to *C*(**x**) or *F*(**x**), ensuring that when combinations of **r** correspond to path binary values and **d** corresponds to discrete flow values, they match the cost or flow values of the respective paths.

The coefficients of fitting *C*(**x**) are shown as an example to illustrate the fitting process using the least squares regression method. Let **θ** be the vector of parameters to be determined, and the fitting error is represented by the loss function *L*(**θ**) as:

(7) $$L(\theta )=||A\theta -C|{|}_{2}^{2}$$

where **A** is the observation matrix, consisting of all variable terms corresponding to the path-flow combinations, **C** is the corresponding cost vector for each combination. To obtain the optimal parameter θ*, we calculate the gradient of the loss function with respect to **θ** and set it to zero:

(8) $$\nabla_{\theta }L(\theta )=2A^{T}A\theta -2A^{T}C=0$$

The analytical solution of the parameter vector is obtained as:

(9) $$\theta^{*}={\left(A^{T}A\right)}^{-1}A^{T}C$$

The matrix **A**^T^**A** is invertible if and only if all columns of the observation matrix **A** are linearly independent, which means there is no perfect multi-collinearity among regression terms. In our construction, the columns of **A** are composed of binary variables for path encoding, flow level encoding, bias coefficient encoding, and auxiliary variables. These groups of variables are mutually independent, as they correspond to disjoint sets of qubits, and the HOBO to QUBO transformation does not introduce linear dependencies among the original variables. In addition, the total number of samples, i.e., the row count of **A**, equals the total number of valid combinations of paths, flow levels, and bias coefficients, which is far larger than the number of regression terms. Under general conditions, distinct binary combinations correspond to distinct cost or flow values, so the columns of **A** are linearly independent, which guarantees the non-singularity of **A**^T^**A** and yields a unique least-squares solution. Meanwhile, penalty coefficients are adopted to constrain invalid path branches instead of directly eliminating the corresponding variables, which further avoids linear correlation between the column vectors of matrix **A**.

Once θ* is obtained, the linear and quadratic coefficients can be derived, which is expressed as:

(10) $$\begin{array}{l} h_{c,k}=\theta_{c,k}^{*},\;\;\;\;h_{c,i}=\theta_{c,i}^{*},\\ J_{c,k_{\alpha }k_{\beta }}=\theta_{c,k_{\alpha }k_{\beta }}^{*},\;\;\;\;J_{c,i_{\alpha }i_{\beta }}=\theta_{c,i_{\alpha }i_{\beta }}^{*},\;\;\;\;J_{c,ki}=\theta_{c,ki}^{*}\\ c_{c}=\theta_{c,\mathrm{last}}^{*} \end{array}$$

where *k_α_*, *k_β_*, *i_α_*, *i_β_* denote the subscripts of different paths and flow levels for coefficients *r* and *d*, respectively. *α* and *β* serve as subscripts to distinguish distinct binary variables when fitting quadratic coefficients, c in the subscript represents the coefficient of cost functions, the fitted values of *h* and *J* correspond to the values of matrices **q_C_** and **Q_C_** in Equation (6), and θ*_last_ is the value of the last column of θ*.

It is worth noting that when representing paths using combinations of *K* binary variables, if 2*^K^ *> *p*, the excess binary combinations correspond to physically infeasible paths. To prevent the optimizer from selecting these infeasible paths during evolution, it is necessary to introduce a penalty term to *C*(**x**), which is expressed as:

(11) $$C^{\prime }(x)=C(x)+\mu_{N}\sum_{k^{\prime }\in S_{N}}r_{k^{\prime }}$$

where *S_N_* denotes the set of invalid path indexes and *k*′ is the index of an invalid path. *μ_N_* is a relatively large penalty coefficient. The pseudocode for fitting coefficients using the least squares method is provided in Appendix B.

To quantitatively evaluate the fitting precision of the least-squares regression for constructing QUBO cost and flow matrices, two standard regression evaluation metrics are introduced: root mean square error (RMSE) and coefficient of determination R^2^. The RMSE regression is defined as [54]:

(12) $$R_{M}=\sqrt{\frac{1}{M}\sum_{m=1}^{M}{\left(y_{m}-{\hat{y}}_{m}\right)}^{2}}$$

where *M* denotes the total number of path-flow sample combinations, *y_m_* represents the real cost or flow value of the *m*-th sample, and ${\hat{y}}_{m}$ is the fitted value reconstructed from solved QUBO linear and quadratic coefficients θ*. Smaller RMSE indicates a smaller overall deviation between the fitted QUBO model and original objective function. The coefficient of determination is calculated as:

(13) $$R^{2}=1-\frac{\sum_{m=1}^{M}{\left(y_{m}-{\hat{y}}_{m}\right)}^{2}}{\sum_{m=1}^{M}{\left(y_{m}-\bar{y}\right)}^{2}}$$

where $\bar{y}$ is the average value of all real sample data. The range of R^2^ is [0, 1]. A value close to 1 implies the QUBO model can accurately capture the non-linear coupling between path variables and flow levels, while a value near 0 indicates severe fitting distortion.

The penalty term *μ_N_* introduced in Equation (11) constrains invalid path combinations generated by redundant binary encoding states. The magnitude of *μ_N_* affects the energy gap between feasible solutions and unphysical invalid path solutions within the QUBO Hamiltonian, thereby altering the sampling probability distribution of the QAOA circuit. If *μ_N_* is set too small, invalid path states retain low energy values and compete effectively with genuine feasible solutions during variational optimization, causing the QAOA algorithm to converge to meaningless invalid routing schemes. Conversely, an excessively large *μ_N_* creates an over-stiff penalty energy landscape that may compress the numerical energy differences among all valid paths, weakening the model’s capability to distinguish the optimal flow-path combinations under various bias coefficients.

To quantify how penalty strength impacts overall optimization performance, a set of gradient values of *μ_N_* are configured for penalty sensitivity simulations in subsequent sections. The RMSE and R^2^ metrics for QUBO coefficient fitting are calculated for each parameter group. By horizontally comparing the fitting accuracy corresponding to different penalty coefficients, we screen out suitable *μ_N_* values that simultaneously satisfy three criteria: full suppression of invalid paths, distinguishable energy gradients among all feasible routes, and low fitting error.

The cost function *C*(*r*,*d*) is expressed as:

(14) $$\begin{array}{l} C(r,d)=\sum_{k}h_{c,k}r_{k}+\sum_{i}h_{c,i}d_{i}+\sum_{k_{\alpha }<k_{\beta }}J_{c,k_{\alpha }k_{\beta }}r_{k_{\alpha }}r_{k_{\beta }}+\\ \;\;\;\;\;\;\;\;\;\;\;\;\;\;\;\sum_{i_{\alpha }<i_{\beta }}J_{c,i_{\alpha }i_{\beta }}d_{i_{\alpha }}d_{i_{\beta }}+\sum_{k,i}J_{c,ki}r_{k}d_{i}+\mu_{N}\sum_{k^{\prime }\in S_{N}}r_{k^{\prime }} \end{array}$$

Similarly, the QUBO expression of the flow function *F*(*r*,*d*) can be obtained through the same fitting process, which is expressed as:

(15) $$\begin{array}{l} F(r,d)=\sum_{k}h_{f,k}r_{k}+\sum_{i}h_{f,i}d_{i}+\sum_{k_{\alpha }<k_{\beta }}J_{f,k_{\alpha }k_{\beta }}r_{k_{\alpha }}r_{k_{\beta }}+\\ \;\;\;\;\;\;\;\;\;\;\;\;\;\;\;\sum_{i_{\alpha }<i_{\beta }}J_{f,i_{\alpha }i_{\beta }}d_{i_{\alpha }}d_{i_{\beta }}+\sum_{k,i}J_{f,ki}r_{k}d_{i}+\mu_{N}\sum_{k^{\prime }\in S_{N}}r_{k^{\prime }} \end{array}$$

Through the above fitting, the effects of path selection and flow level have been uniformly represented as combinations of linear and quadratic terms of binary variables. However, in multi-objective optimization, the cost and flow terms still need to be balanced through bias coefficient.

In this paper, *λ* in Equation (2) is also introduced as an optimization variable into the QUBO framework and is modeled in a discrete form. This discretization process can be consistent with the binary representations of the previous path and flow variables. *λ* is uniformly divided into *B* discrete levels. To efficiently encode these *B* discrete levels, a set of binary variables *a* = {*a*_1_, *a*_2_,…, *a_Y_*} are introduced, where *Y* = ⌈log_2_ *B*⌉. The discrete values of *λ* are represented using binary weighted summation, which is expressed as:

(16) $$\lambda (a)=\lambda_{\min}+\frac{\lambda_{\max}-\lambda_{\min}}{B-1}\sum_{j=0}^{Y}2^{-j}a_{j}$$

Through the above representation, *λ* is no longer a continuous bias coefficient but a discrete bias coefficient uniquely determined by a set of binary variables.

Next, we couple *λ*(*a*) with the binary path-flow variables (*r*, *d*) to modulate the weight of the flow and cost fitting term. The coupled objective function is given by (17):

(17) min    H(a,r,d)=λ(a)C(r,d)−(1−λ(a))F(r,d)

Nevertheless, Equation (15) inevitably involves third-order terms such as *a_j_*·*r_k_*·*d_i_*, thereby yielding a higher-order binary optimization (HOBO) formulation. Current mainstream quantum optimization algorithms only support at most quadratic terms, so the model cannot be directly solved. Therefore, it is necessary to equivalently convert the third-order terms into quadratic forms. A common approach is to introduce an auxiliary binary variable that is constrained to equal the product of two target binary variables, with large positive numbers *G* to enforce this equality [55,56]. Based on the characteristics of (14) and (15), in order to minimize the number of introduced auxiliary variables, we replace the product of *a_j_* and *r_k_* in the HOBO model with the auxiliary binary variable *y_jk_*, *y_jk_* = *a_j_*·*r_k_*, and replace the product of *a_j_* and *d_i_* with the auxiliary binary variable *y_ji_*, *y_ji_* = *a_j_*·*d_i_*. Then, the minimization of the third-order term *a_j_*·*r_k_*·*d_i_* can be equivalently formulated as:

(18) $$\begin{array}{l} \underset{a_{j},r_{k},d_{i},y}{\min}\;\;\;\;y_{jk}d_{i}+\sum_{j,k}G_{1}(3y_{jk}+a_{j}r_{k}-2a_{j}y_{jk}-2r_{k}y_{jk})+\\ \;\;\;\;\;\;\;\;\;\;\;\;\;\;\;y_{ji}r_{k}+\sum_{j,i}G_{2}(3y_{ji}+a_{j}d_{i}-2a_{j}y_{ji}-2d_{i}y_{ji}) \end{array}$$

At last, the complete QUBO model is as follows:

(19) $$\begin{array}{l} \underset{a_{j},r_{k},d_{i},y}{\min}\;\;\;\;\lambda (a)C^{*}(r,d,y)+(1-\lambda (a))F^{*}(r,d,y)+\\ \;\;\;\;\;\;\;\;\;\;\;\;\;\;\;\sum_{j,k}G_{1}(3y_{jk}+a_{j}r_{k}-2a_{j}y_{jk}-2r_{k}y_{jk})+\\ \;\;\;\;\;\;\;\;\;\;\;\;\;\;\;\sum_{j,i}G_{2}(3y_{ji}+a_{j}d_{i}-2a_{j}y_{ji}-2d_{i}y_{ji}) \end{array}$$

where C*(*r*, *d*, *y*) and F*(*r*, *d*, *y*) denote the quadratic versions of *C*(*r*,*d*) and *F*(*r*,*d*) after replacing all *a_j_*·*r_k_*·*d_i_* terms with *y_jk_d_i_* or *y_ji_r_k_*, the decision variables are [*r*_1_,…, *r_K_*, *d*_1_,…, *d_I_*, *a*_1_,…, *a_Y_*, *y*_11_,…, *y_jk_*, *y_ji_*]. Equation (19) conforms to the standard QUBO form and is directly solvable by quantum optimization algorithms.

Based on the RMSE and R^2^ evaluation metrics established above, we carry out sensitivity analysis to reveal the influence of fitting bias of the least-squares surrogate model on the optimization outputs of the QAOA framework. Since the fitting residuals of least-squares regression follow a zero-mean Gaussian distribution, Gaussian noise is injected into the QUBO formulation to simulate different degrees of fitting distortion. Specifically, independent white Gaussian noise is added to the linear coefficient vectors **q_C_**, **q_F_** and symmetric quadratic matrices **Q_C_**, **Q_F_**, respectively. The standard deviation of noise is taken as the control variable to construct three distortion scenarios: mild, moderate, and severe. Finally, the corresponding evaluation metrics under these three scenarios are calculated.

The probability density function of a single Gaussian noise element is given by:

(20) $$p(\delta )=\frac{1}{\sqrt{2\pi }\sigma }\exp(-\frac{\delta^{2}}{2\sigma^{2}})$$

where *δ* is a single random noise term added to QUBO coefficients, simulating the residual deviation of least-squares fitting, and *σ* is the controllable standard deviation serving as the hyperparameter to adjust noise intensity. The statistical property E[*δ*^2^] = *σ*^2^ indicates that the average fluctuation amplitude of noise *δ* rises linearly with the growth of *σ*.

After injecting Gaussian noise *δ***_θ_** into the original coefficient vector θ*, the disturbed coefficient vector is θ’ = θ* + δ_θ_. The predicted target value of the m-th sample becomes ${\hat{y}}_{m}$ = **A_m_θ’**. Under the fixed regression matrix A, the mathematical expectation of RMSE has a linear proportional relationship with *σ*:

(21) $${E[R}_{M}]=\sqrt{\frac{1}{M}\sum_{m=1}^{M}A_{m}\cdot A_{m}^{T}}\cdot \sigma$$

where **A_m_** is the m-th row extracted from the complete regression matrix **A**. Larger *σ* leads to higher expected RMSE, which means the overall absolute deviation between the surrogate model and real objective values increases.

The detailed experimental procedure for analyzing the sensitivity of optimization results to fitting errors is described as follows. First, the noise-free benchmark RMSE and R^2^ are calculated using the original QUBO coefficients fitted by the least-squares algorithm as reference criteria. Next, three noise intensity groups corresponding to low, moderate, and high standard deviations are configured. All Gaussian random disturbances *δ* follow the distribution *δ*∼N(0,*σ*^2^) and are superimposed on the symmetric quadratic matrices **Q_C_**, **Q_F_** and linear vectors **q_C_**, **q_F_**. Afterwards, the predicted values of all samples are reconstructed based on the disturbed QUBO coefficients, and the corresponding RMSE and R^2^ of each group are recalculated. A larger value of *σ* leads to a greater amplitude of random disturbance δ, which corresponds to a higher RMSE and a lower R^2^.

After establishing the QUBO model, the number of binary variables is *O*(*Y*(*K* + *I*)), where *K* = ⌈log_2_ *P*⌉, *I* = ⌈log_2_ *L*⌉, and *Y* = ⌈log_2_ *B*⌉. In contrast, classical enumeration requires traversing all discrete states. Therefore, the logarithmic encoding proposed in this paper achieves an exponential compression of the search space in representation, with the number of required qubits growing logarithmically with the number of classical candidate states. It is important to emphasize that an exponential reduction in the number of variables does not inherently equate to computational speedup or provable quantum advantage. Instead, it demonstrates that the problem can be encoded with drastically fewer quantum resources, which is a prerequisite for solving large-scale instances on near-term quantum devices. The final solution is obtained by extracting the combination of binary variables that minimizes the objective function for each bias coefficient, which yields the selected paths and their corresponding flow levels.

### 2.2. Solution Process of the Built Quantum Optimization Model

The above optimization model exhibits a one-to-one mapping to the Ising Hamiltonian and can be directly embedded into the QAOA framework for solving. QAOA searches for the optimal solution in the superposition state space through a parameterized quantum gate sequence, which naturally biases the probability distribution toward low-energy states, thereby obtaining paths with the lowest cost under different bias coefficients.

The QAOA achieves a global search driven by quantum interference by alternately applying a cost Hamiltonian and a mixing Hamiltonian within the quantum state space. We suppose the QUBO model contains *N* binary decision variables, which correspond to *N* qubits, where each qubit represents a decision variable that takes values only in {0, 1}. During initialization, a Hadamard gate is applied to all qubits to generate a uniform superposition state, which is expressed as:

(22) $$|\varphi_{0}\rangle =H^{⊗N}{|0\rangle }^{⊗N}$$

This state covers all possible path-flow combinations in parallel within the solution space, providing an initial state for the global search. The cost Hamiltonian can be constructed as follows:

(23) $$H_{C}=\sum_{i}\kappa_{i}Z_{i}+\sum_{i<j}\gamma_{ij}Z_{i}Z_{j}$$

where *Z_i_* and *Z_j_* denote the Pauli-Z operator, and the coefficients are derived from the QUBO model via the standard mapping: *γ_ij_* = 1/4*Q_ij_* and *κ_i_* = −1/2*q_i_* − 1/4∑ _*j* ≠ *i*_*Q_ij_*, where *q_i_* and *Q_ij_* correspond to the entries of matrix **q** and **Q** in Equation (6) of the QUBO model, with the binary variables *x_i_* ∈ {0, 1} mapped to the Pauli-Z operators via *x_i_* = (1 + Z*_i_*)/2. QAOA minimizes the expectation value of *H_C_* over the parameterized variational ansatz using a classical optimizer, thereby approximating the ground state energy of the cost Hamiltonian, whose corresponding eigenstate encodes the optimal solution to the original path-flow optimization problem.

First, the number of QAOA layers *s_l_* is specified, and the parameter vector is initialized using a uniform distribution, which is expressed as:

(24) $$\gamma_{k}^{\left(0\right)}∼U(0,\pi ),\;\;\;\;\beta_{k}^{\left(0\right)}∼U(0,\frac{\pi }{2})$$

During the initialization of variational parameters, all rotation angles *γ* and *β* corresponding to the evolution of the cost Hamiltonian and mixing Hamiltonian are sampled uniformly at random within the interval [0, 2π]. Proper random initialization prevents the optimization process from being trapped in shallow local minima at the early stage of iterations and ensures full exploration of the parameter space of variational angles.

Next is the quantum state evolution. Given the parameters (*γ*, *β*), the QAOA circuit is constructed. Each layer first applies the cost evolution operator:

(25) $$U_{C}(\gamma_{k})=e^{-i\gamma_{k}H_{C}}$$

where the linear terms are implemented using single-qubit R_Z_ gates, while the quadratic coupling terms are encoded via R_ZZ_ gates or an equivalent CNOT–R_Z_–CNOT structure. Subsequently, the mixing evolution operator *U_M_* is applied:

(26) $$U_{M}(\beta_{k})=e^{-i\beta_{k}H_{M}},H_{M}=\sum_{i=1}^{n}X_{i}$$

where *H_M_* is the mixing Hamiltonian; this corresponds to applying an Rx rotation gate to each qubit.

After alternately applying the above two types of operators, the quantum state with layer *s_l_* is expressed as [57]:

(27) $$|\varphi_{s_{l}}(\gamma ,\beta )\rangle =\prod_{k=1}^{s_{l}}U_{M}(\beta_{k})U_{C}(\gamma_{k}){|+\rangle }^{⊗N}$$

The circuit depth of QAOA, represented by *s_l_*, interacts restrictively with the total number of qubits. A larger qubit count corresponds to a higher-dimensional Hilbert space, which demands more variational layers to complete exhaustive solution space searching. Conversely, fewer qubits only require a smaller number of layers. The logarithmic qubit encoding adopted in this paper restricts qubit overhead to a logarithmic scale relative to the number of paths, weakening the positive correlation between network scale and circuit depth and drastically slowing the growth of circuit depth under scenarios with massive candidate paths. In Qiskit Aer simulations, the qubit count and variational layers should be coordinately configured to balance the exploration capability over the solution space and the time cost of simulation. Matching an excessive qubit set with overly deep layers introduces massive quantum gates and extends the sampling time, while equipping abundant qubits with insufficiently shallow layers results in incomplete searching and biased output solutions.

The subsequent step involves measurement and the evaluation of the expected energy. We perform measurements on the quantum state with respect to the computational basis {|*x_i_*⟩}_{0, 1}_. This measurement procedure is executed *N_s_* times to accumulate a collection of samples. Denoting the frequency of the state |*x_i_*⟩ as *N_x_*, the estimated probability is given by:

(28) $$P(x)=\frac{N_{x}}{N_{s}}$$

Each measurement sample *x* corresponds to a complete path-flow combination scheme, and based on the sampled probabilities, the expected energy under the current variational parameters (*γ*, *β*) is evaluated as:

(29) $$E(\gamma ,\beta )=\sum_{x}P(x)C_{v}(x)$$

where *C_v_*(*x*) represents the cost value corresponding to this solution. As the expected energy *E*(*γ*,*β*) decreases gradually, the probability amplitudes of the quantum states converge to the path-flow combinations with low energy, enabling the approximation of the optimal solution.

A classical optimizer is used to iteratively update the parameters *γ* and *β*, such as:

(30) $${\left(\gamma ,\beta \right)}^{\left(t+1\right)}={\left(\gamma ,\beta \right)}^{\left(t\right)}-\eta \nabla E$$

where *η* is the learning rate. The algorithm terminates when the change in expected energy or the gradient satisfies the convergence condition. After the algorithm converges, the final state of the quantum system can be written as:

(31) $$|\varphi^{*}\rangle =\sum_{x}\alpha_{x}|x\rangle ,\;\;\;\;\sum_{x}{\left|\alpha_{x}\right|}^{2}=1$$

**Figure 2:**
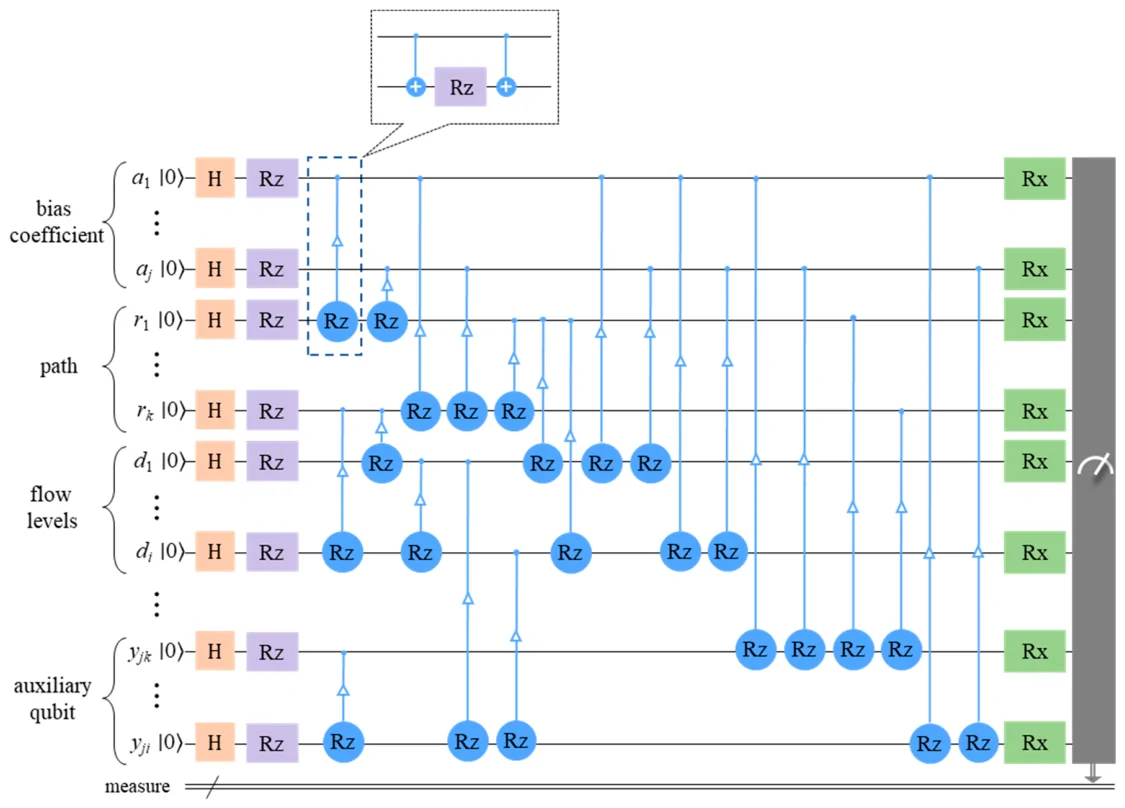
Schematic of the QAOA quantum circuit for the built quantum optimization model. The blocks of different colors represent different quantum gates, and the lines with arrows indicate the combination of three quantum gates. The circuit is divided into four parts, corresponding to qubits for bias coefficients, paths, flow levels, and auxiliary qubits, respectively. Initially, the Hadamard gate is applied to each qubit to generate a uniform superposition state. Then, the Rz gate is applied to each qubit, together with the CNOT–Rz–CNOT structure between every pair of qubits. For simplicity, these three quantum gates are combined and represented as the shape shown in the figure, corresponding to the cost evolution operator, i.e., the linear and quadratic terms of the QUBO model. Since the values corresponding to the bias coefficient qubits *a_j_* increase linearly, there is no CNOT–Rz–CNOT structure between these *a_j_* qubits, while such internal structures exist in the qubits of the other parts. Next, an Rx gate is applied to each qubit, corresponding to the mixing evolution operator. Finally, measurement is performed.

After performing multiple measurements on this state, the occurrence frequency of the path-flow combination |*x*⟩ satisfies *P*(*x*) → |*α_x_*|^2^. The quantum circuit diagram of each layer of QAOA is shown in Figure 2.

Finally, the optimal values corresponding to each bias coefficient were obtained by QAOA, including the paths and flow discretization levels. The quantum model described above can serve as a global coarse screening tool for single-path flow optimization. If the bias coefficient and flow level are highly discretized, the solution from QAOA could be treated as the optimal solution. Or we can further refine the flow and cost as addressed in the next subsection.

### 2.3. Fine-Tuning Based on Cubic Spline Interpolation

After QAOA completes the global screening of discrete path-flow combinations, the optimal path and its corresponding flow level can be determined. The original flow interval [0, *f_p_*_,max_] has been narrowed to a local flow interval Γ centered on the estimated flow level:

(32) $$\Gamma =[f_{p,i}-\frac{f_{p,\max}}{2(L-1)},f_{p,i}+\frac{f_{p,\max}}{2(L-1)}]$$

where *f_p_*_,*i*_ is the screened result of QAOA for the flow level *i* of path *p*.

In practical networks, cost and flow cannot be directly expressed as continuous functions. To address this issue, this paper constructs a continuously differentiable approximation function based on discrete samples to characterize the overall variation trend of cost with flow. Before introducing cubic spline interpolation for refined optimization, this work proposes the following prerequisites. The global cost-flow function may contain non-smooth points caused by link capacity saturation, yet QAOA-based coarse optimization limits the search range to a local interval Γ. This setup reduces the likelihood that global abrupt variations and non-smooth regions lie inside this interval. Discrete sampling performed within the local interval allows collected samples to fully capture the curve’s non-linear changes. Even when minor non-smooth features appear on the original curve, every piecewise cubic polynomial only fits a narrow local segment of the curve. As a result, the fitted function precisely matches local curve shapes without noticeable deviations. In addition, natural boundary conditions with zero second derivatives at both endpoints are utilized. This scheme removes the requirement of manually assigning boundary derivative values and circumvents extra restrictive assumptions about global smoothness.

To this end, cubic spline interpolation is adopted in this paper to fit the discrete flow-cost relationship. In particular, we select *W* discrete sample points over the interval Γ, denoted as (*f_ε_*,*c_ε_*(*f*)) with *ε* = 1,2, …, *W*. A piecewise cubic polynomial is constructed on each subinterval formed by adjacent sample points, with continuity constraints imposed on the function value, first derivative, and second derivative. Meanwhile, natural boundary conditions S″(*f*_1_) = S″(*f_W_*) = 0 are introduced. Accordingly, the objective function is obtained as S(*f*) = *λC*(*p*) + (1−*λ*)*F*(*p*). When *f* belongs to the interval [*f_ε_*, *f_ε_*_+1_], we use the cubic spline interpolation function to obtain a part of *S*(*f*), which is denoted as *S_ε_*(*f*):

(33) $$S_{\varepsilon }(f)=\rho +\varsigma (f-f_{\varepsilon })+\xi {\left(f-f_{\varepsilon }\right)}^{2}+\zeta {\left(f-f_{\varepsilon }\right)}^{3},\;\;\;\;\;\;\;\;\;f\in [f_{\varepsilon },f_{\varepsilon +1}]$$

where *ρ*, *ς*, *ζ*, and *ξ* are coefficients. The interpolation conditions are *S_ε_*(*c*(*f*)) = *f_ε_* and *S_ε_*_+1_(*c*(*f*)) = *f_ε_*_+1_. The first-order continuity condition is *S*′*_ε_*_+1_(*c*(*f*)) = *f*′_ε+1_, and the second-order continuity condition is *S*″*_ε_*_+1_(*c*(*f*)) = *f*″_ε+1_.

Subsequently, solving for the optimal flow is transformed into an extreme-value analysis problem for the piecewise cubic spline function. Since *C*(*f*) is constructed by a natural cubic spline, we have *S*(*f*) ∈ **C**^2^(Γ), and its minimum value can be determined by derivative conditions. On each subinterval [*f_ε_*, *f_ε+_*_1_], *S*(*f*) is a cubic polynomial whose first derivative is a quadratic function. The extreme points satisfy *S*′(*f*) = 0, and this equation yields at most two real roots in each subinterval. Only real roots lying within the corresponding subinterval are retained as candidate points, and *S*″(*f*) > 0 is used to verify that they are local minima. Meanwhile, the function values at the interval endpoints *f*_1_ and *f*_M_ are compared to avoid missing boundary optimal solutions. If multiple local minima exist, their objective function values are compared uniformly, and the smallest one is selected as the global optimum. The set of possible optimal solutions is given by:

(34) $$F_{S}=\{f_{1},f_{M}\}\cup \{f|S^{\prime }(f)=0,f\in \Gamma ,S^{\prime \prime }(f)>0\}$$

In summary, the cubic spline uses piecewise cubic polynomials to construct a continuous cost-flow function, extending the discrete optimization results into an analytically tractable continuous form. While achieving exact interpolation, this method ensures the continuity of the first and second derivatives and uniquely determines the spline coefficients through natural boundary conditions to obtain a smooth and stable optimal solution.

## 3. Results

**Figure 3:**
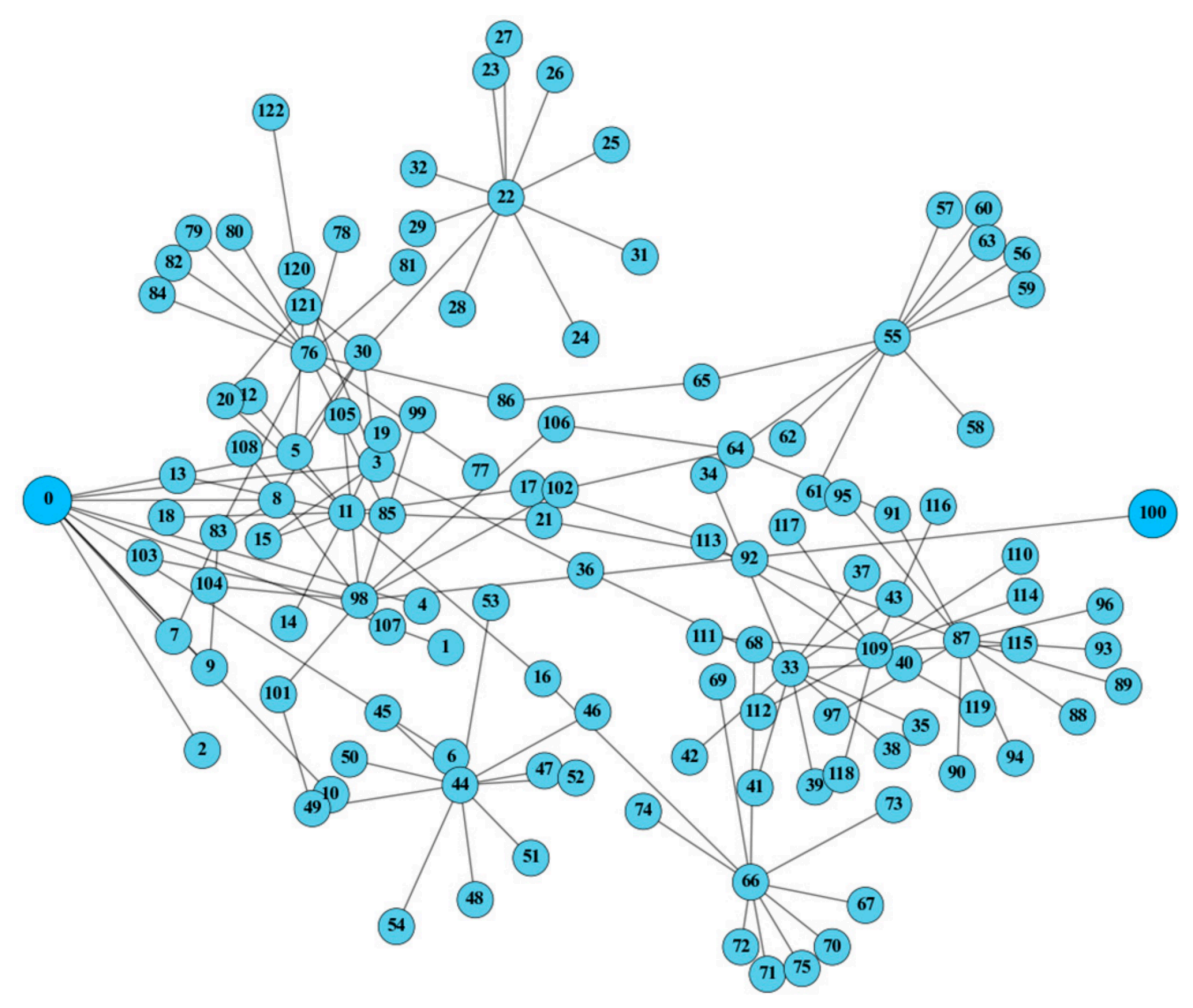
Schematic diagram of the network with 122 nodes and 131 edges. We transmit flow from Node 0 to Node 100. It can be observed that each node in this network is connected to others via only a few edges, exhibiting the characteristics of a small-world network. The large number of nodes and edges results in a total of 82 paths from the source node 0 to the destination node 100.

We take an Internet of things (IoT) communication network with 122 nodes and 131 edges as the test case to verify the performance of the proposed method [58]. The topology of this network is illustrated in Figure 3. A total of 5000 units flow is transmitted from Node 0 to Node 100. Calculated by the DFS algorithm, there are a total of *p* = 82 flow transmission paths between the two nodes, the flow capacity range of all single paths is [110, 1100], and the cost range covers [10.557, 98.835]. The raw dataset is provided in Appendix C.

In this numerical example, we focus on the single-source and single-sink scenario, where optimization is performed under different bias coefficients to obtain the minimum cost for transmitting a specified amount of flow. The feasibility and computational advantages of the proposed method are evaluated using indicators such as encoding scale, qubit requirement, objective function convergence, as well as the final performance in terms of cost.

First, the binary encoding scheme for decision variables is specified as follows. A total of *K* = ⌈log_2_ 82⌉ = 7 qubits are adopted to encode the 82 valid paths, and the remaining 46 binary index combinations correspond to invalid paths. For flow level encoding, the cost-flow values are evenly discretized into *L* = 16 levels represented by *I* = 4 qubits. This configuration is designed to clearly visualize experimental outcomes and prevent indistinct optimization results caused by excessive qubit consumption. Regarding the encoding of cost-flow bias coefficients, the feasible interval of *λ* is set to [0.25, 0.75], which is also uniformly discretized into *B* = 16 levels encoded with *Y* = 4 qubits.

To quantitatively evaluate the fitting accuracy of the high-order least-squares regression model for constructing QUBO coefficients, we perform parametric sensitivity simulations of the invalid-path penalty coefficient *μ_N_* on this 82-node topology, with the RMSE and coefficient of determination R^2^ adopted as unified quantitative metrics. Three gradient representative values *μ_N_* = {5, 10, 100} are selected to cover low, medium, and high penalty intensities. For each value of *μ_N_*, the complete QUBO coefficient matrix is reconstructed via the least squares method, and the corresponding fitting indicators RMSE and R^2^ are calculated to conduct penalty sensitivity analysis. All statistical results are summarized in Table 1.

As shown in the table, the distortion of the model fitting is slightly larger when the penalty coefficient is set to a small value of *μ_N_* = 5. The root cause is that the ineffective binary path encoding state has not been adequately suppressed, which leads to high sampling probabilities for physically meaningless paths. When *μ_N_* is set to 10 and 100, the RMSE and R^2^ metrics reach nearly ideal values; these values can effectively eliminate the encoding of invalid paths while distinguishing all feasible routes. Overall, the fitting accuracy remains acceptable for all penalty coefficients no less than 5.

**Figure 4:**
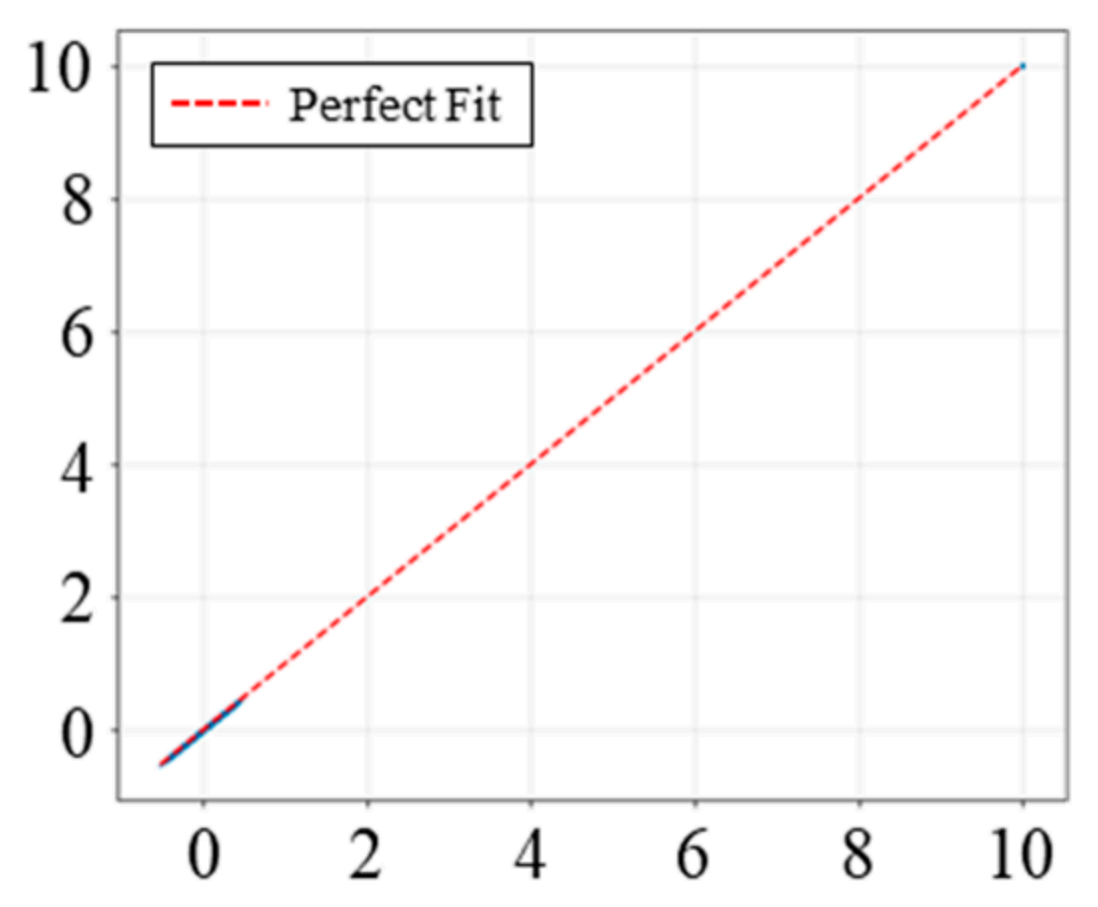
The fitting curve of QUBO coefficients obtained via the least-squares method. The blue scatter points correspond to the true values and predicted values calculated by the objective function *H*(*p*,*f_p_*) in Equation (2), and the red dashed line denotes the perfect fitting reference line.

The least-squares model can accurately approximate the true objective function of the network path-flow optimization problem, with global fitting residuals maintained at an extremely low level. Therefore, *μ_N_* = 10 is adopted as the penalty coefficient for all subsequent experiments. Figure 4 presents the scatter fitting plot comparing the true objective values and the model-predicted values under *μ_N_* = 10.

Sample points are densely distributed along the fitted diagonal line, demonstrating an excellent consistency between regression fitting outputs and raw sample data. Minor deviations of a small number of sample points stem from inherent tiny residuals of polynomial regression. Such subtle fitting errors will not introduce evident bias into the subsequently constructed QUBO Hamiltonian and only exert limited slight impacts on the final optimization results solved by the QAOA framework. Furthermore, the subsequent cubic spline interpolation algorithm can mitigate this effect.

**Figure 5:**
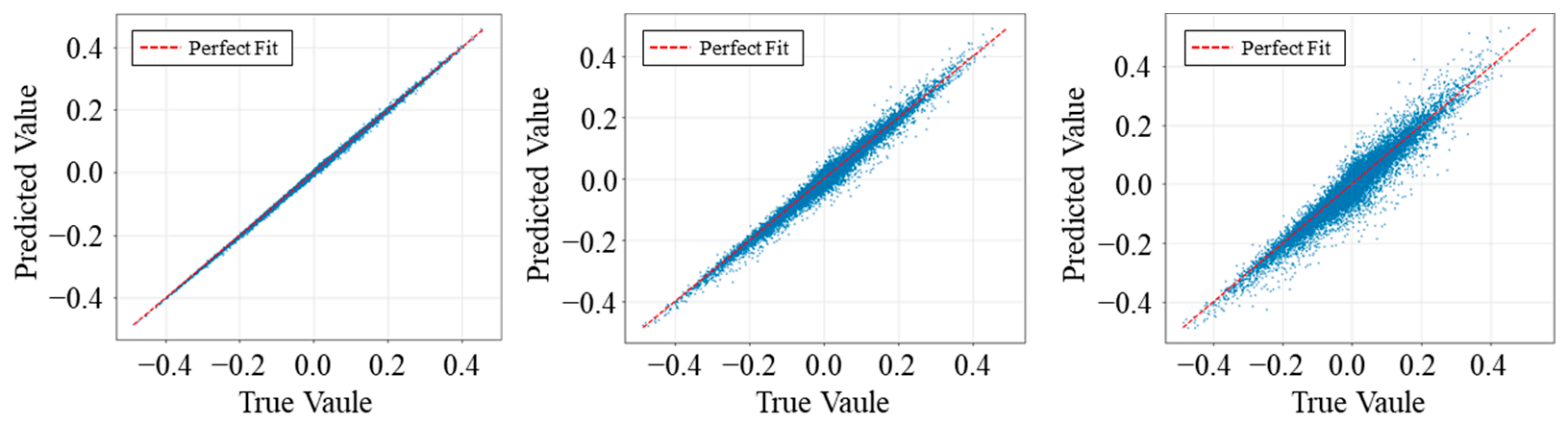
The influence diagram of different standard deviation Gaussian noises (from left to right, the Gaussian noises with standard deviations of 0.0002, 0.001, and 0.002, respectively) on the fitting error. To clearly visualize the fitting errors, these figures omit data points with large penalty values and only display values ranging from −0.4 to 0.4.

To further quantify the sensitivity of the least-squares fitting model to coefficient perturbations and explore the influence of fitting distortion on the subsequent QAOA optimization performance, three groups of Gaussian white noise with different intensities are added to the fitted QUBO coefficient matrices and vectors in this paper. The standard deviations of the three groups of noise are set to σ = 0.0002, σ = 0.001, and σ = 0.002, corresponding to mild, moderate, and severe fitting distortion conditions, respectively. Each group of noise follows a zero-mean Gaussian distribution *δ*∼N(0,*σ*^2^) and is superimposed on all linear and high-order polynomial coefficients. After noise injection, the regression predicted values of all samples are reconstructed, and the corresponding RMSE and R^2^ metrics are recalculated. The indicators under each noise level are summarized in Table 2, and the comparison plots of true values versus predicted values for each noise case are presented in Figure 5.

The RMSE increases monotonically with the growth of noise intensity, while R^2^ decreases gradually simultaneously. Under mild disturbance with σ = 0.0002, the fitting accuracy remains at a relatively high level, with an RMSE of 0.0031 and an R^2^ of 0.9991, which indicates that tiny fitting residuals barely degrade the representation capability of the surrogate model. When the noise intensity rises to the moderate level of *σ* = 0.001, the RMSE climbs to 0.0155 and R^2^ drops to 0.9775, revealing that moderate fitting distortion introduces observable deviations to the predicted objective values. In the case of severe disturbance at *σ* = 0.002, the RMSE reaches 0.0309 and R^2^ further declines to 0.9099, demonstrating that the fitting model generates an obvious deviation, which will reduce the reliability of the constructed QUBO model. When Gaussian noise is added to simulate fitting deviation, the energy landscape of *H*(*p*,*f_p_*) will be deformed. Mild errors only slightly adjust local fluctuations of the landscape, while moderate and severe errors alter the energy ranking of all feasible solutions and shift the true global minimum of the original optimization problem. Since QAOA minimizes the energy of QUBO Hamiltonian as the optimization objective, distortion of energy landscape will directly interfere with parameter optimization of variational circuits.

We perform QUBO coefficient fitting on the combination of the above qubits and then conduct computation using QAOA. The number of variational layers is calibrated collaboratively as *s_l_* = 8 according to the total number of qubits and the scale of 82 feasible paths. This moderate configuration balances the full exploration capability of the solution space and the computational overhead of simulations on the Qiskit Aer platform. The Adam gradient descent optimizer is adopted in the classical optimization module to iteratively update the variational rotation angles *γ* and *β*, with a fixed learning rate of 0.02 and a gradient clipping threshold of 0.1 to stabilize parameter updates. The iteration terminates when the absolute change of the cost expectation falls below 10^−5^. In the measurement sampling phase, 10^5^ repeated measurement shots are performed for each set of variational parameters to reduce statistical noise. All tests are carried out on the ideal statevector simulator of Qiskit Aer without hardware noise interference. During variational parameter initialization, the initial rotation angles *γ* and *β* are sampled uniformly at random within the range [0, 2π]. The numerical simulations in this work employ a global uniform random initialization scheme without artificial bias, and a fixed random seed of 42 is set as the core reproducibility parameter.

**Figure 6:**
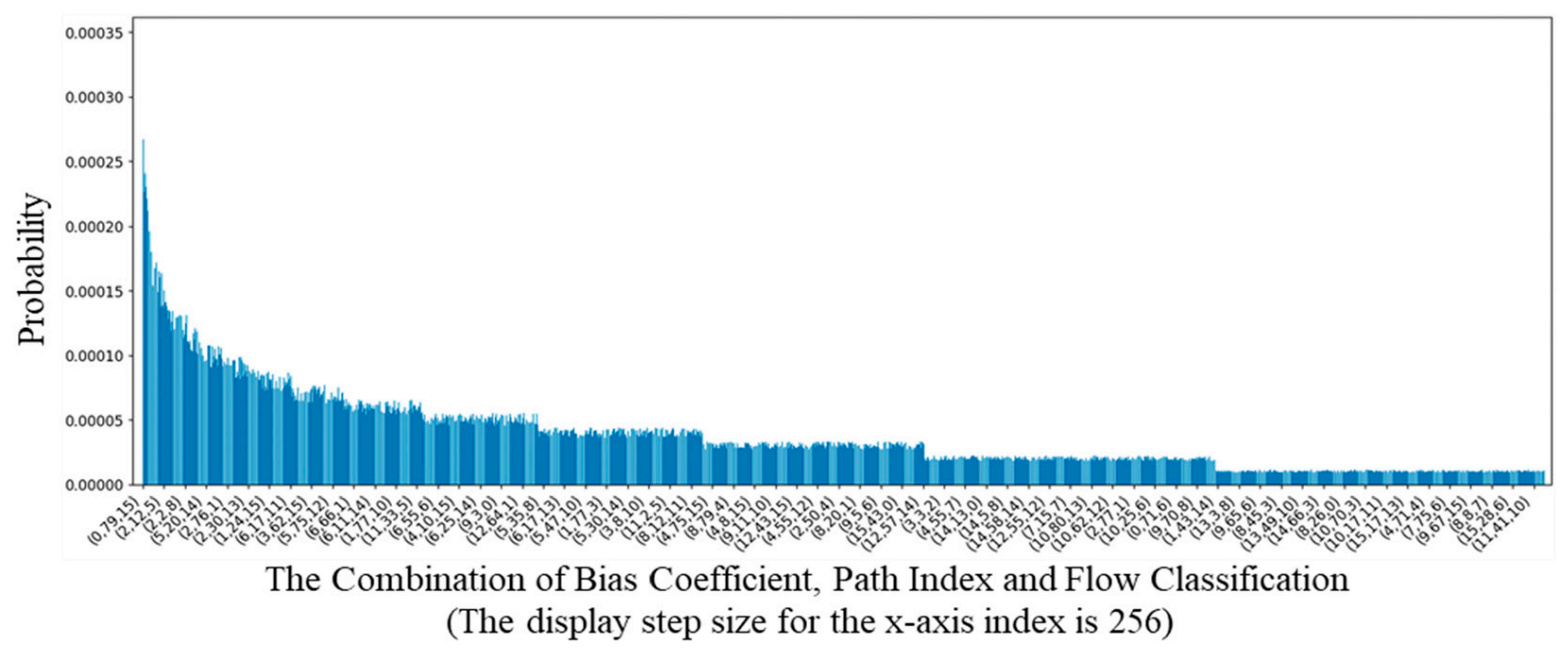
Optimization results of path selection and flow level under different bias coefficients using the QAOA algorithm. The three values in the brackets of the horizontal coordinate, respectively, represent the discrete index of the bias coefficient *λ*, the path index *r*, and the discrete level index *d* of path flow. For display convenience, the step size of the horizontal axis is set to 256, and a total of 67 indices are listed on the x-axis. The combinations with higher probabilities indicate that the optimizer prefers these solutions.

Figure 6 shows the calculation results of the QAOA algorithm. The horizontal axis represents the binary values of all qubits. For better presentation, the horizontal axis is converted into decimal form in the order of bias coefficient *λ*, path index *r*, flow level *d*. The auxiliary qubits that couple the bias coefficient qubits with the path index qubits and flow level qubits, respectively, are not shown in the figure since they have no actual physical meaning.

**Figure 7:**
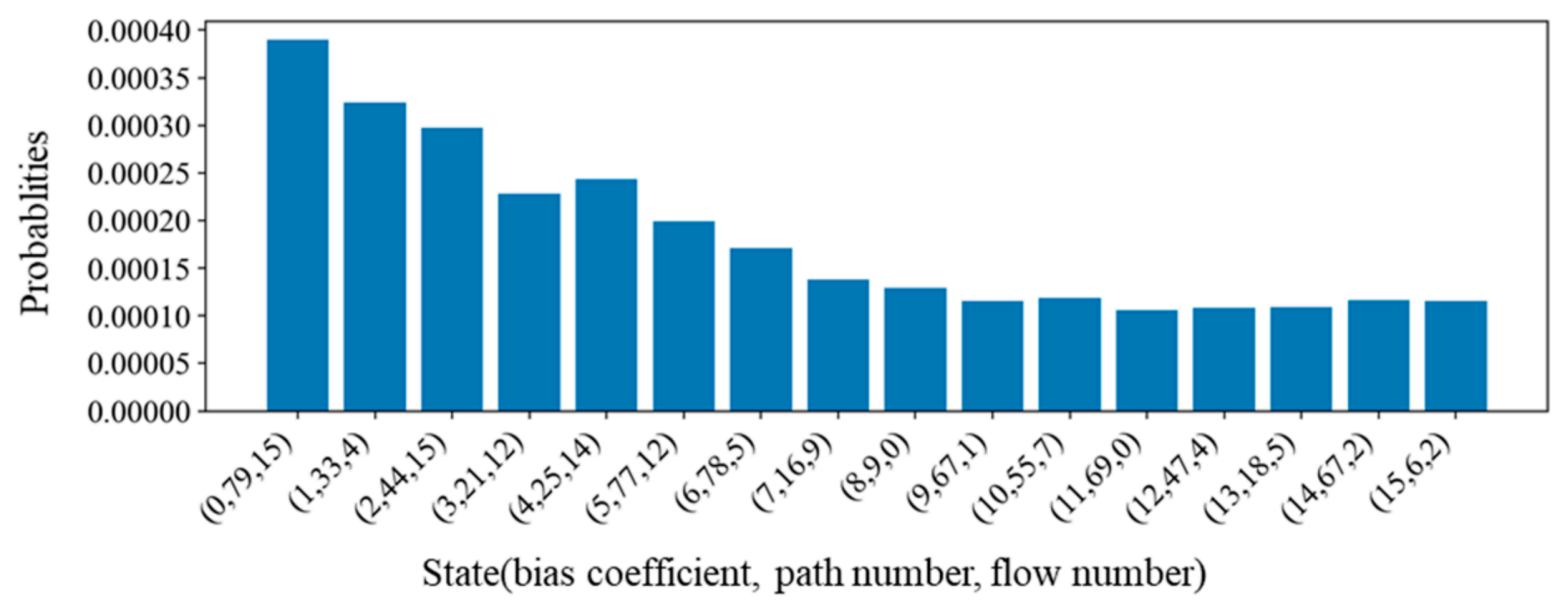
Optimal values of path-flow-level combinations under different bias coefficients.

After obtaining the measurement outputs of the QAOA circuit, all sampled quantum states are grouped according to the qubit vector which encodes the bias coefficient *λ*. Each group uniquely corresponds to one discrete value of *λ*. Within each group with a fixed *λ*, we select the sampled state that yields the minimum Hamiltonian energy; this state stores the optimal discrete path index and discrete flow level under the corresponding *λ*, and these grouped optimal results are visualized in Figure 7.

Subsequently, we extract the optimal path, coarsely optimized flow, and transmission cost for all 16 discrete λ values. Taking the flow bounds of 110 and 1000 as the benchmark, which are the minimum and maximum value of transmission flow, respectively, normalized flow values are converted back to physical real values. The actual flow volume and total transmission cost under a total throughput demand of 5000 units are then calculated and summarized in Table 3.

Comparison reveals that the minimum total cost is achieved at *λ* = 0.6833, with Path 18 selected and a discrete flow value of 230. It can be observed that several results in Table 3 exhibit close magnitudes. Furthermore, the preliminary solutions obtained by QAOA contain numerical biases arising from model fitting errors and inherent algorithmic deviations of QAOA itself. To address this issue, we adopt the cubic spline interpolation algorithm to perform precise continuous optimization within the narrowed local flow interval. Piecewise polynomial fitting is utilized to correct numerical offsets of discrete flow outputs, yielding the exact minimum cost under each λ and refining the final optimal solutions.

**Figure 8:**
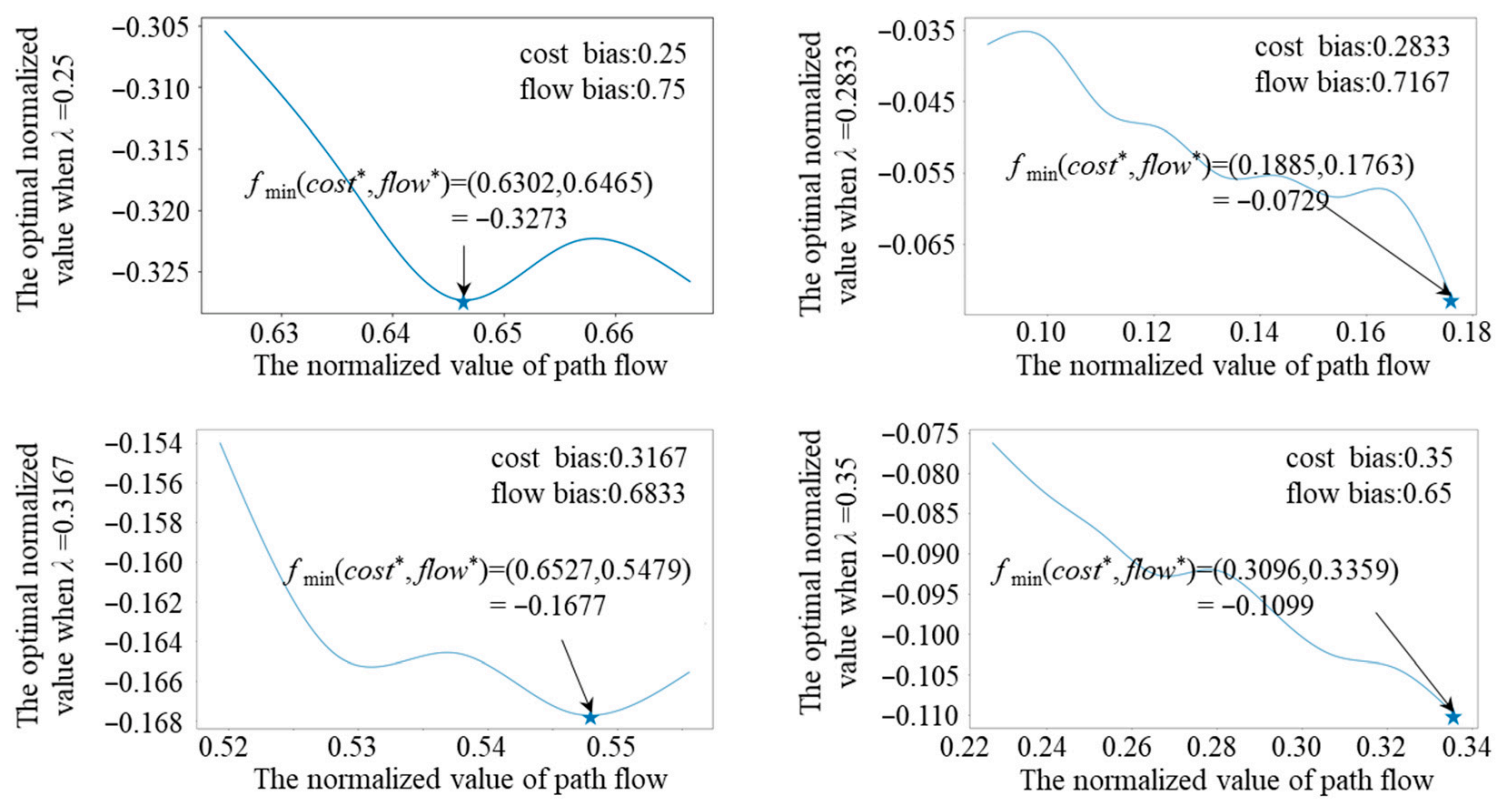

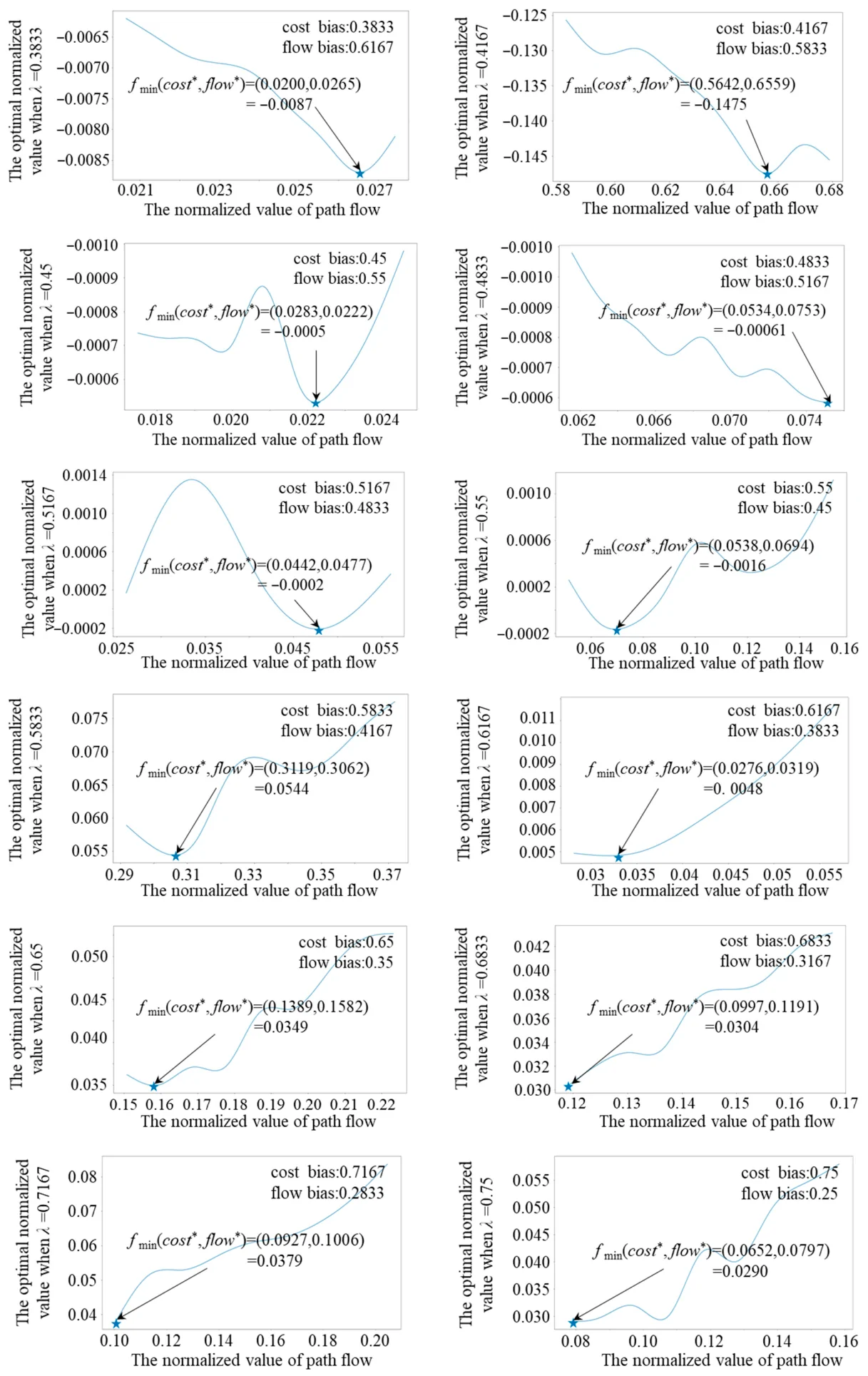
Spline interpolation optimizations under different bias coefficients. Star symbols denote the points where the objective function reaches its minimum value. The horizontal axis represents the normalized flow, and the vertical axis represents the optimized values denoted as S(*f*). The top-right corner of each subplot indicates the current bias coefficients.

When implementing cubic spline interpolation, if the corresponding flow obtained by quantum optimization falls at the minimum or maximum flow level (i.e., 0 or 15), we sample the flow values corresponding to 4 different flow sub-levels in the sub-intervals Γ, otherwise, we sample 8 flow sub-levels. We fit these sampling points using spline interpolation functions under different bias coefficients, yielding a total of 16 optimization results, as shown in Figure 8.

The normalized values in Figure 8 need to be restored to the exact values of the optimal path and the corresponding cost and flow for different bias coefficients *λ*, as listed in Table 4.

It can be observed that the total cost for each bias coefficient decreases noticeably after refinement. In addition, for a transmission flow of 5000 units, it can be observed that the comprehensive cost shows a general downward trend as *λ* increases. The minimum comprehensive cost is not obtained at the maximum bias coefficient, but at *λ* = 0.6833, where the minimum value of comprehensive cost is 448.104. This further demonstrates that in the topology of this numerical example, the cost of flow transmission is affected by the non-linear relationship between cost and flow.

To verify the accuracy and analytical performance advantages of the logarithmic qubit-encoded QAOA combined with cubic spline interpolation optimization framework proposed in this paper, two mainstream classical optimization algorithms in the path scheduling field are adopted for comparative experiments, including mixed integer programming (MIP) as an exact algorithm and genetic algorithm (GA) as a heuristic algorithm. The optimization results of MIP are taken as the benchmark to evaluate the solution accuracy and computational overhead of the proposed method, while GA serves as an auxiliary comparison object for further verification. Specifically, the solution accuracy is quantified by the deviation between the optimal objective values obtained by different algorithms. The computational overhead is measured by the total number of iterative steps consumed by each algorithm to finish all optimization tasks covering 16 groups of bias coefficients *λ* ranging from 0.25 to 0.75.

**Figure 9:**
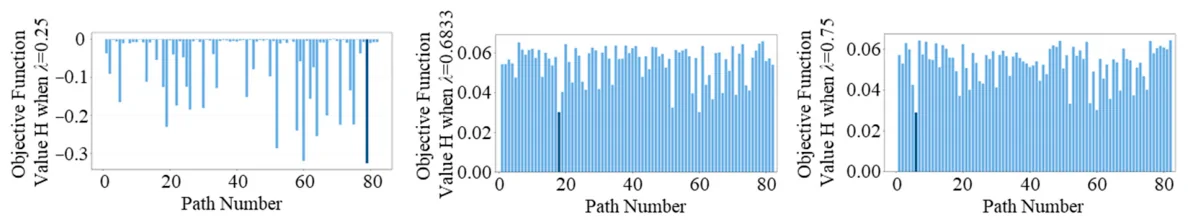
The optimization result of the MIP algorithm.

Figure 9 presents the bar charts of optimization results under three typical cases where the bias coefficient *λ* takes the lower bound 0.25, the upper bound 0.75, and 0.6833. The objective function values of all 82 paths are calculated. When the bias coefficient *λ* = 0.6833, the transmission cost for 5000 flow units reaches the minimum, and this case achieves the optimal trade-off between flow and cost. The bar charts intuitively reveal obvious differences in the objective function values among different paths, enabling clear identification of the optimal scheduling path corresponding to each trade-off coefficient.

In the figure, dark bars indicate the optimal paths achieving the minimum objective function. The horizontal axis is the path index, and the vertical axis is the objective function value. For bias coefficient *λ* = 0.25, the optimal path is 79, the minimum objective function value is −0.3257, and the transmission cost for 5000 flow units is 531.4898. For *λ* = 0.6833, the optimal path is 18, the minimum objective function value is 0.0302, and the transmission cost for 5000 flow units is 461.5532; this *λ* corresponds to the minimum cost. For *λ* = 0.75, the optimal path is 6, the minimum objective function value is 0.029, and the transmission cost for 5000 flow units is 463.0017.

**Figure 10:**
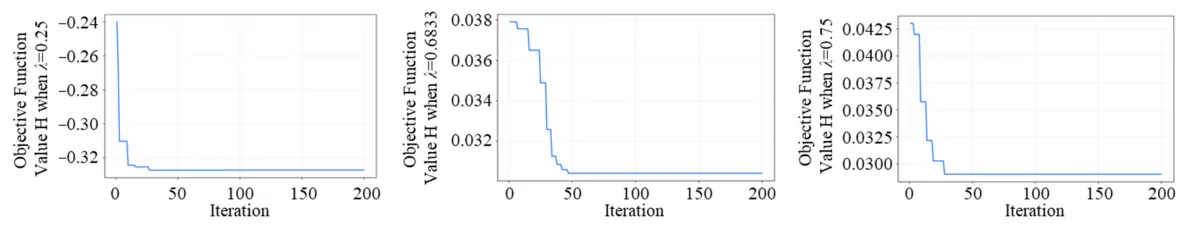
The iterative process diagram of GA.

For comparison, GA is adopted to solve the same problem. The hyperparameters are set as follows: population size *pop_size* = 120, maximum iteration *gen_max* = 200, crossover probability *pc* = 0.72, mutation probability *pm* = 0.14, and tournament size *tour_size* = 6. The iterative optimization curves are illustrated in Figure 10.

The horizontal axis of the figure represents the iteration number, and the vertical axis denotes the objective function value. As observed from the iterative curves, the objective function value of the genetic algorithm continuously declines and gradually stabilizes with ongoing iterations. The algorithm basically converges within 50 generations under all three test cases. Nevertheless, the genetic algorithm requires independent iterative optimization for each bias coefficient. In complex multi-path optimization scenarios, it incurs higher computational overhead and tends to converge to local optimum.

When the bias coefficient *λ* = 0.25, the optimal path is 79, the minimum objective function value is −0.3274, and the transmission cost for 5000 flow units is 528.4473. When *λ* = 0.6833, the optimal path is 18, the minimum objective function value is 0.0304, and the transmission cost for 5000 flow units is 461.8759; this *λ* corresponds to the lowest cost. When *λ* = 0.75, the optimal path is 6, the minimum objective function value is 0.029, and the transmission cost for 5000 flow units is 463.2557.

It can be observed that for the test case with 122 nodes and 82 candidate paths, the proposed QAOA and cubic spline interpolation algorithm achieves objective function results with an error of 0.547% compared with the exact optimization algorithm MIP and an error of 0.601% compared with the GA.

The aggregated results of all numerical comparison sufficiently demonstrate that the proposed hybrid optimization framework exhibits excellent solution accuracy. In terms of accuracy, the discrete flow solutions from QAOA are refined by cubic spline interpolation. The obtained optimal paths, accurate transmission flows, and global comprehensive costs are in high agreement with the benchmark global optimal solutions of exact MIP, and the average relative error is kept below 1%.

In terms of computational overhead and solution workflow, we investigate 16 groups of bias coefficient scenarios covering the entire cost-flow trade-off interval. As an exact enumerative algorithm, MIP demands separate modeling for different bias coefficients and exhaustive exploration of all feasible paths. The overall computational complexity increases with the number of paths and bias coefficient groups, which tends to cause computational bottlenecks in large-scale networks. As a mainstream heuristic optimization algorithm, the GA also requires independent full iterative runs for each bias coefficient. It depends on random population search to converge to approximate solutions progressively, incurring a large total iteration count and prolonged computation time per scenario.

In contrast, the logarithmic-qubit encoded QAOA optimization scheme proposed in this paper embeds all bias coefficients, candidate paths, and discrete flow levels into a unified QUBO Hamiltonian model. It only requires one-time construction of the quantum model to simultaneously compute optimization results under different *λ* scenarios. During the variational solving phase, a shallow QAOA quantum circuit with fixed layers and a small set of variational parameters is adopted for iterative optimization. Leveraging quantum superposition, the algorithm traverses the entire solution space consisting of all path-flow-bias combinations in parallel. After only a few rounds of classical gradient updates, the discrete optimal combinations corresponding to all bias coefficients can be obtained upon convergence. The subsequent refinement module based on cubic spline interpolation incurs low computational cost, which merely performs limited sampling and polynomial fitting within the narrow local flow interval preliminarily screened by QAOA. The overall workflow balances accuracy and computational efficiency and can yield optimization results comparable to MIP and GA. It demonstrates distinctive computational advantages for single-path scheduling scenarios in complex networks.

To further compare the performance of the proposed optimization framework combining QAOA and cubic spline interpolation with classical optimization algorithms in terms of solution accuracy and computational efficiency, we investigate a network topology consisting of 8000 nodes. There are 7586 transmission paths from node 1 to node 8000, with a flow range of [70, 1540] and a cost range of [6.528, 157.248]. Under this setup, *K* = 13 qubits are required to encode path indices. Flow values are discretized into 16 levels, which demands *I* = 4 qubits. The feasible range of *λ* is set to [0.25, 0.75] and evenly split into 16 discrete levels, corresponding to *Y* = 4 qubits. The variational depth of QAOA is configured as 10, and all remaining hyperparameters are consistent with those adopted in the previous test case featuring 82 paths. Similarly, MIP and GA are introduced as comparative benchmarks, and their corresponding optimization results are summarized in Table 5.

As illustrated by the comparison results in Table 5, none of the three algorithms (MIP, GA, and the proposed quantum optimization framework) can guarantee strictly global optimal outputs when the network scale expands drastically. Although the selected transmission paths and optimized objective values differ among the three algorithms in the large-scale 8000 nodes test case, the overall error of all schemes relative to the theoretical optimum is controlled within 5%. The discrepancy in path selection arises primarily because single-path scheduling does not require operating at each path’s maximum capacity, and multiple distinct paths can bear similar flow loads. In addition, the values of bias coefficient *λ* that yield the minimum transmission cost slightly vary across algorithms, yet all deviations fall within a reasonable and acceptable range.

The comparison results of the two sets of test cases reveal that for the small-scale network instance, the total transmission cost and optimal paths solved by the proposed optimization framework are highly consistent with those generated by the classical exact MIP algorithm and heuristic GA algorithm, with an overall relative error of less than 1%. For the large-scale test scenario, the increased topological complexity and sharp surge in the number of feasible paths lead to obvious discrepancies between the optimal solutions yielded by the two traditional algorithms (MIP and GA). Nevertheless, the relative error between the results solved by our framework and the optimal values from traditional algorithms is steadily maintained within 5%. The above two tests fully verify that the proposed optimization framework achieves stable and reliable solution accuracy for both small- and medium-sized networks as well as ultra-large-scale network scenarios.

## 4. Discussion

This paper proposes a hybrid quantum optimization framework that integrates logarithmic qubit encoding, QAOA, and cubic spline interpolation for single-path routing in complex networks. Path selection, flow variables, and cost-flow bias coefficients are uniformly modeled as a QUBO model with logarithmic-scale decision variables. Least squares regression is employed to transform the optimization parameters into standard QUBO coefficients, and auxiliary binary variables are introduced to eliminate high-order coupling terms while quantifying fitting errors. The bias coefficients are embedded into a single energy function in the form of encodable qubits, avoiding redundant modeling. After global coarse screening by QAOA, the classical numerical method of natural cubic spline interpolation is adopted to refine discrete quantum solutions and generate precise optimization results.

To conduct comprehensive numerical validation, two topological test cases are constructed: a small IoT network with 122 nodes and a large-scale network with 8000 nodes, with MIP and GA selected as benchmark algorithms for comparison. For the 122 nodes test case, the relative error of the proposed scheme against the global optimum obtained by traditional optimization algorithms is less than 1%. Even for the large-scale test case, the overall error of our method remains within an acceptable range. Compared with conventional optimization algorithms, the proposed method has several distinct advantages. The logarithmic encoding drastically reduces qubit consumption, and only one round of quantum variational optimization is required to simultaneously output optimal solutions corresponding to all bias coefficients. The cubic spline interpolation can effectively compensate for the precision loss caused by QAOA. Comparative experiments on the two test cases fully demonstrate that the proposed optimization framework achieves outstanding solution accuracy, generalization capability, and computational efficiency for networks of various sizes.

## Figures and Tables

**Table 1:** Model fitting accuracy under different penalty coefficients *μ_N_*.

Penalty *μ_N_*	RMSE	R^2^
5	0.0230	0.9957
10	0.0018	0.9999
100	0.0018	0.9995

**Table 2:** Fitting accuracy under different Gaussian noise intensities.

Noise Intensity	Standard Deviation *σ*	RMSE	R^2^
Noise-free (benchmark)	0	0.0018	0.9999
Low	0.0002	0.0031	0.9991
Medium	0.001	0.0155	0.9775
High	0.002	0.0309	0.9099

**Table 3:** QAOA optimization results under different bias coefficients *λ*.

λ	Path	Optimal Flow	Total Cost When the Transmission Flow Is 5000
0.25	79	700	529.1429
0.2833	33	262	516.9656
0.3167	44	620	566.4435
0.35	21	414	495.7166
0.3833	25	138	507.8623
0.4167	77	654	493.0693
0.45	78	126.667	512.2095
0.4833	16	176	502.9356
0.5167	9	110	484.4091
0.55	67	155.333	490.5625
0.5833	55	385.333	505.5502
0.6167	69	110	474.8636
0.65	47	256.667	464.2652
0.6833	18	230	460.4699
0.7167	67	200.667	483.3801
0.75	6	182	461.4671

**Table 4:** Fine-tuning optimization results under different bias coefficients *λ*.

λ	Path	Optimal Flow	Total Cost When the Transmission Flow Is 5000
0.25	79	685.385	524.867
0.2833	33	266.907	514.485
0.3167	44	597.631	550.385
0.35	21	408.951	490.234
0.3833	25	133.585	495.242
0.4167	77	693.751	489.048
0.45	78	129.758	497.052
0.4833	16	177.017	495.343
0.5167	9	149.783	482.665
0.55	67	171.766	485.548
0.5833	55	382.518	487.898
0.6167	69	138.391	469.431
0.65	47	250.798	454.928
0.6833	18	215.999	448.104
0.7167	67	199.534	469.594
0.75	6	180.933	450.802

**Table 5:** The optimization results of the three algorithms in the large-scale topology.

λ	Metrics	MIP ^1^*	GA ^2^*	QAOA ^3^* & Cubic Spline Interpolation
0.25	Path selection	3574	5819	6147
	Optimal objective value	−0.0263	−0.0214	−0.0251
	Optimal flow	495.881	518.409	506.532
	Total cost (flow = 5000)	535.877	518.273	526.497
0.75	Path selection	1845	953	4759
	Optimal objective value	0.0355	0.0360	0.0302
	Optimal flow	196.597	216.955	189.332
	Total cost (flow = 5000)	429.501	453.874	416.579
*λ* for min total cost (flow = 5000)		0.7167	0.6833	0.6833
	Path selection	4952	1339	5871
	Optimal objective value	0.0312	0.0299	0.0327
	Optimal flow	234.189	257.995	284.507
	Total cost (flow = 5000)	420.057	431.596	414.278

^1^* Mixed integer programming; ^2^* Genetic algorithm; ^3^* Quantum approximate optimization algorithm.

## Data Availability

This theoretical simulation study does not generate new experimental data. All supporting path data, network parameters and algorithm codes are provided in the manuscript and Appendix A, Appendix B and Appendix C.

## Abbreviations

The following abbreviations are used in this manuscript:

QUBO	Quadratic Unconstrained Binary Optimization	
QAOA	Quantum Approximate Optimization Algorithm	
QA	Quantum Annealing	
DFS	Depth First Search	
RMSE	Root Mean Square Error	
HOBO	Higher-Order Binary Optimization	
IoT	Internet of Things	
MIP	Mixed Integer Programming	
GA	Genetic Algorithm	
*G*(*V*,*E*)	Directed connected network, Graph (nodes,edges)	
(*m*,*n*) ∈ *E*	Two nodes connecting on edge *E*	
*u_mn_*	Maximum capacity of the directed edge (*m*,*n*)	
*c_mn_*	Unit flow cost of the directed edge (*m*,*n*)	
s	Starting node	
t	Ending node	
*P*	Number of feasible paths from s to t	
*H*	Objective function	
*f_p_*	Flow value of the *p*-th path	
*F*(*p*)	The total transmission flow of a single path in the objective function	
*C*(*p*)	The total transmission cost of the single path in the objective function	
*f_p_*_,max_	Maximum flow of the *p*-th path	
*E_p_*	The edges on *p*-th path	
*λ*	Cost-flow bias coefficient	
*λ*_min_, *λ*_max_	Lower and upper limit of *λ*	
*c*_min_, *c*_max_	Minimum and maximum value of cost	
*f*_min_, *f*_max_	Minimum and maximum value of flow	
*c**	Normalized dimensionless cost variable	
*f**	Normalized dimensionless flow variable	
*F*	Total transmission flow	
*K*	Number of qubits for path encoding	
*r_k_*	Binary variable for path encoding	
*L*	Number of discrete levels for the maximum flow of a path	
*I*	Number of qubits required for flow level encoding	
*d_i_*	Binary variable for flow level encoding	
**x**	Combined binary variable vector for path and flow encoding	
*C*(**x**)	Fitted cost value from binary variables	
*F*(**x**)	Fitted flow value from binary variables	
***Q_C_***, ***Q_F_***	Symmetric matrix of quadratic coefficients for cost and flow fitting	
***q_C_***, ***q_F_***	Linear coefficient vector for cost and flow fitting	
***c_C_***, ***c_F_***	Constant term vector for cost and flow fitting	
*S_N_*	Set of invalid path indices	
*k*′	Index of an invalid path	
*μ_N_*	Penalty coefficient	
R_M_	Root mean square error metric calculation	
*M*	Total number of path-flow sample combinations	
*y_m_*	Real cost or flow value of the *m*-th sample	
${\hat{y}}_{m}$	Fitted value reconstructed from solved QUBO linear and quadratic coefficients	
R^2^	Coefficient of determination metric calculation	
$\bar{y}$	Average value of all real sample data	
*p*(*δ*)	Probability density function of a single Gaussian noise element	
*δ*	A single random noise term	
*σ*	Controllable standard deviation	
**δ*_θ_***	Gaussian noise injected into coefficient vector	
**θ′**	Disturbed coefficient vector after adding Gaussian noise	
**A_m_**	The m-th row extracted from the complete regression matrix	
E[*δ*^2^]	Mean squared fluctuation amplitude of Gaussian noise	
E[R_M_]	Mathematical expectation of the RMSE over all M samples	
N(0,σ^2^)	Gaussian normal distribution followed by random fitting noise	
*L*(**θ**)	The loss function of the least squares method	
**A**	Observation matrix formed by the decision vectors	
***C***	Cost vector corresponding to path-flow combinations	
**θ***	Expanded parameter vector	
**θ***_last_	Value of the last column of the parameter vector **θ***	
*k_α_*, *k_β_*, *i_α_*, *i_β_*	Subscripts of different paths and flow levels in the linear and quadratic coefficients of binary variables	
*h*	Linear coefficient of binary variables	
*J*	Quadratic coefficient of binary variables	
*C*(*r*,*d*)	Path cost represented by binary variables and coefficient	
*F*(*r*,*d*)	Path flow represented by binary variables and coefficient	
*B*	Number of discrete levels for *λ*	
*Y*	Number of qubits required for *λ*	
*a_j_*	Binary variable for bias coefficient encoding	
*λ*(*a*)	Discretized bias coefficient represented by *a_j_*	
*y_jk_*, *y_ji_*	Auxiliary binary variables	
*G*,*G*_1_,*G*_2_	Large positive numbers of the conversion from HOBO to QUBO	
*N*	Total number of binary decision variables in the QUBO model	
*Z_i_*, *Z_j_*	Pauli-Z operator	
*γ**_ij_*	Quadratic coefficient in cost Hamiltonian	
*κ_i_*	Linear coefficient in cost Hamiltonian	
*x_i_*	Generalized binary variables	
*s_l_*	Number of layers of QAOA	
*γ*, *β*	Parameters of QAOA	
*U_M_*	Mixing evolution operator	
*H_M_*	Mixing Hamiltonian	
*Ns*	Number of measurements of ∣*x*⟩	
*Nx*	Number of occurrences of ∣*x*⟩	
*P*(x)	Estimated occurrence probability of ∣*x*⟩	
*C_v_*(x)	Cost value corresponding to the sample x	
*E*(*γ*, *β*)	Expected energy of the quantum state	
*η*	Learning rate	
*τ*	Iteration index	
*α_x_*	Amplitude of ∣*x*⟩	
Γ	Local optimization interval	
(*f_ε_*,*c_ε_*(*f*))	Discrete sample points selected within the interval Γ	
*W*	Number of discrete sample points	
*S*(*f*)	Objective function constructed by cubic spline interpolation	
*C*(*f*)	Cost function constructed by cubic spline interpolation	
*ρ*,*ς*,*ζ*,*ξ*	Coefficients of the piecewise cubic polynomial	
*S_ε_*(*f*)	Piecewise cubic polynomial on the *ε*-th subinterval	
*F_S_*	The set of possible optimal solutions	
*pop_size*	Population in GA algorithm	
*gen_max*	Maximum iteration in GA algorithm	
*pc*	Crossover probability in GA algorithm	
*pm*	Mutation probability in GA algorithm	
*tour_size*	Tournament size	

## Appendix A

**Algorithm A1:** Formal Description of the Single-Path Flow Transmission Problem
Input: A directed or undirected network G = (V, E) with edge capacities *u_ij_*, transmission costs *c_ij_*, source node s, sink node t, and flow demand F. Output: An optimal single-path flow decision minimizing total transmission cost under flow constraints. Initialize: Define the feasible path set *P_s_*_→_ *_t_*, which *p* is a simple path from *s* to *t* Step 1: Formalization of the Cost–Flow Model def DFS(current_node): Add current_node to CurrentPath Mark current_node as visited if current_node == t: Add a copy of CurrentPath to PathList else: For each outgoing edge (current_node, j) in E: If j not in Visited: DFS(j) Remove current_node from CurrentPath Unmark current_node as visited Step 2: Path Capacity Evaluation For each path p in PathList: PathCapacity ← +∞ For each consecutive node pair (*i*, *j*) in p: PathCapacity ← min(PathCapacity, *u_ij_*) Assign PathCapacity to path p Step 3: Path Cost Calculation For each path p in PathList: PathCost ← 0 For each consecutive node pair (*i*, *j*) in p: PathCost ← PathCost + *c_ij_* Assign PathCost to path p Step 4: Optimal Path Selection If PathCost(p) < BestCost: BestCost ← PathCost(p) BestPath ← p Result A formalized single-path flow transmission model with explicit cost–flow bias coefficient representation, together with a systematic identification of computational limitations in classical optimization methods.

## Appendix B

**Algorithm A2:** Pseudocode for Least-Squares Fitting of First and Second Order Binary Coefficients
Input: Binary variable samples *X*, target values *y*, coefficient truncation threshold *epsilon* Output: Fitted first-order coefficients *h_i_*, fitted second-order coefficients *J_ij_*, constant term *const* Initialize: Define an empty list *Regression*Terms to store all regression components, define an empty list *CoefficientLabels* to record the physical meaning of each term Step 1: Construct regression terms # First-order linear terms For each var in BinaryVars: Append var to RegressionTerms, Append “FirstOrder_” + var.label to CoefficientLabels # Second-order interaction terms (unordered pairs) For each var1, var2 in BinaryVars where var1 ≠ var2 and (var2,var1) ∉ PairSet: Add (var1,var2) to PairSet Append var1×var2 to RegressionTerms Append SecondOrder + var1.label + var2.label to CoefficientLabels # Constant bias term Append ConstantBias to RegressionTerms Append “ConstantBias_Term” to CoefficientLabels Step 2: Solve linear regression system # Stack regression terms column-wise to form the design matrix regression_matrix ← column_stack (RegressionTerms) # Solve the overdetermined system using least squares to minimize fitting error coefficient_vector ← least_squares_solve(regression_matrix, target_values) Step 3: Coefficient Truncation and Sparsification # Apply thresholding to filter negligible coefficients predefined_threshold ← user_specified_value # Predefined threshold for sparsity for idx in 0 to len(coefficient_vector) −1: coeff = coefficient_vector[idx] if abs(coeff) < predefined_threshold: coefficient_vector[idx] ← 0 # Zero out negligible coefficients for sparsity Step 4: Output of the Binary Optimization Model # Extract non-zero coefficients and constant term nonzero_coeffs ← filter(coeff ≠ 0 for coeff in [first_order_coeffs, second_order_ coeffs]) constant_term ← constant_bias_term # Form and return quadratic binary model for QUBO optimization quadratic_binary_model ← compose(nonzero_coeffs, constant_term) return (nonzero_coeffs, constant_term) Result A compact quadratic binary model obtained via least-squares regression, capturing dominant linear and pairwise interactions among binary variables, and directly applicable to classical or quantum optimization frameworks.

## Appendix C

All paths from Node 0 to Node 100 are listed in Table A1 below, including all nodes traversed and the maximum allowable flow. Due to the non-linearity of the unit flow cost, the total flow of each path is presented here.

**Table A1:** Raw data table.

Path	The Nodes that the Path Passes Through	*f_p_*_,max_	Total *c_p_*
1	0-3-15-11-20-121-5-30-8-83-76-86-65-55-64-102-98-100	720	83.606
2	0-3-15-11-20-121-5-30-8-83-76-86-65-55-64-106-98-100	480	51.423
3	0-3-15-11-20-121-30-8-83-76-86-65-55-64-102-98-100	440	51.267
4	0-3-15-11-20-121-30-8-83-76-86-65-55-64-106-98-100	570	49.549
5	0-3-15-11-21-92-87-91-64-102-98-100	460	46.347
6	0-3-15-11-21-92-87-91-64-106-98-100	650	58.407
7	0-3-30-5-121-20-11-21-92-87-91-64-102-98-100	380	45.161
8	0-3-30-5-121-20-11-21-92-87-91-64-106-98-100	850	81.140
9	0-3-30-8-83-76-86-65-55-64-102-98-100	800	78.245
10	0-3-30-8-83-76-86-65-55-64-106-98-100	460	42.460
11	0-3-30-121-20-11-21-92-87-91-64-102-98-100	770	66.746
12	0-3-30-121-20-11-21-92-87-91-64-106-98-100	770	71.169
13	0-3-120-121-5-30-8-83-76-86-65-55-64-102-98-100	700	60.962
14	0-3-120-121-5-30-8-83-76-86-65-55-64-106-98-100	660	58.196
15	0-3-120-121-20-11-21-92-87-91-64-102-98-100	410	34.659
16	0-3-120-121-20-11-21-92-87-91-64-106-98-100	220	21.455
17	0-3-120-121-30-8-83-76-86-65-55-64-102-98-100	770	83.032
18	0-3-120-121-30-8-83-76-86-65-55-64-106-98-100	470	39.653
19	0-5-30-3-15-11-21-92-87-91-64-102-98-100	750	72.734
20	0-5-30-3-15-11-21-92-87-91-64-106-98-100	820	83.045
21	0-5-30-3-120-121-20-11-21-92-87-91-64-102-98-100	490	47.360
22	0-5-30-3-120-121-20-11-21-92-87-91-64-106-98-100	420	51.631
23	0-5-30-8-83-76-86-65-55-64-102-98-100	950	91.521
24	0-5-30-8-83-76-86-65-55-64-106-98-100	480	60.341
25	0-5-30-121-20-11-21-92-87-91-64-102-98-100	140	14.517
26	0-5-30-121-20-11-21-92-87-91-64-106-98-100	520	50.659
27	0-5-30-121-120-3-15-11-21-92-87-91-64-102-98-100	790	75.028
28	0-5-30-121-120-3-15-11-21-92-87-91-64-106-98-100	960	89.014
29	0-5-121-20-11-15-3-30-8-83-76-86-65-55-64-102-98-100	440	43.845
30	0-5-121-20-11-15-3-30-8-83-76-86-65-55-64-106-98-100	160	14.811
31	0-5-121-20-11-21-92-87-91-64-102-98-100	630	62.948
32	0-5-121-20-11-21-92-87-91-64-106-98-100	370	45.632
33	0-5-121-30-3-15-11-21-92-87-91-64-102-98-100	680	75.267
34	0-5-121-30-3-15-11-21-92-87-91-64-106-98-100	550	57.628
35	0-5-121-30-8-83-76-86-65-55-64-102-98-100	560	47.556
36	0-5-121-30-8-83-76-86-65-55-64-106-98-100	210	22.318
37	0-5-121-120-3-15-11-21-92-87-91-64-102-98-100	150	12.372
38	0-5-121-120-3-15-11-21-92-87-91-64-106-98-100	660	71.288
39	0-5-121-120-3-30-8-83-76-86-65-55-64-102-98-100	400	39.217
40	0-5-121-120-3-30-8-83-76-86-65-55-64-106-98-100	160	17.427
41	0-7-83-8-30-3-15-11-21-92-87-91-64-102-98-100	620	60.850
42	0-7-83-8-30-3-15-11-21-92-87-91-64-106-98-100	330	28.446
43	0-7-83-8-30-3-120-121-20-11-21-92-87-91-64-102-98-100	200	17.036
44	0-7-83-8-30-3-120-121-20-11-21-92-87-91-64-106-98-100	620	70.239
45	0-7-83-8-30-5-121-20-11-21-92-87-91-64-102-98-100	330	28.748
46	0-7-83-8-30-5-121-20-11-21-92-87-91-64-106-98-100	250	23.272
47	0-7-83-8-30-5-121-120-3-15-11-21-92-87-91-64-102-98-100	660	57.589
48	0-7-83-8-30-5-121-120-3-15-11-21-92-87-91-64-106-98-100	350	44.115
49	0-7-83-8-30-121-20-11-21-92-87-91-64-102-98-100	650	55.479
50	0-7-83-8-30-121-20-11-21-92-87-91-64-106-98-100	680	69.817
51	0-7-83-8-30-121-120-3-15-11-21-92-87-91-64-102-98-100	340	30.676
52	0-7-83-8-30-121-120-3-15-11-21-92-87-91-64-106-98-100	680	76.130
53	0-7-83-76-86-65-55-64-102-98-100	850	83.953
54	0-7-83-76-86-65-55-64-106-98-100	170	16.521
55	0-8-30-3-15-11-21-92-87-91-64-102-98-100	700	73.052
56	0-8-30-3-15-11-21-92-87-91-64-106-98-100	750	82.034
57	0-8-30-3-120-121-20-11-21-92-87-91-64-102-98-100	500	41.700
58	0-8-30-3-120-121-20-11-21-92-87-91-64-106-98-100	170	14.386
59	0-8-30-5-121-20-11-21-92-87-91-64-102-98-100	830	81.200
60	0-8-30-5-121-20-11-21-92-87-91-64-106-98-100	750	71.355
61	0-8-30-5-121-120-3-15-11-21-92-87-91-64-102-98-100	530	62.117
62	0-8-30-5-121-120-3-15-11-21-92-87-91-64-106-98-100	120	10.557
63	0-8-30-121-20-11-21-92-87-91-64-102-98-100	430	42.040
64	0-8-30-121-20-11-21-92-87-91-64-106-98-100	400	35.483
65	0-8-30-121-120-3-15-11-21-92-87-91-64-102-98-100	110	12.941
66	0-8-30-121-120-3-15-11-21-92-87-91-64-106-98-100	360	35.185
67	0-8-83-76-86-65-55-64-102-98-100	790	79.149
68	0-8-83-76-86-65-55-64-106-98-100	680	58.285
69	0-9-83-8-30-3-15-11-21-92-87-91-64-102-98-100	550	49.870
70	0-9-83-8-30-3-15-11-21-92-87-91-64-106-98-100	600	59.380
71	0-9-83-8-30-3-120-121-20-11-21-92-87-91-64-102-98-100	360	39.711
72	0-9-83-8-30-3-120-121-20-11-21-92-87-91-64-106-98-100	890	84.963
73	0-9-83-8-30-5-121-20-11-21-92-87-91-64-102-98-100	920	79.204
74	0-9-83-8-30-5-121-20-11-21-92-87-91-64-106-98-100	700	59.131
75	0-9-83-8-30-5-121-120-3-15-11-21-92-87-91-64-102-98-100	430	43.541
76	0-9-83-8-30-5-121-120-3-15-11-21-92-87-91-64-106-98-100	870	76.953
77	0-9-83-8-30-121-20-11-21-92-87-91-64-102-98-100	790	70.954
78	0-9-83-8-30-121-20-11-21-92-87-91-64-106-98-100	160	16.903
79	0-9-83-8-30-121-120-3-15-11-21-92-87-91-64-102-98-100	700	74.080
80	0-9-83-8-30-121-120-3-15-11-21-92-87-91-64-106-98-100	220	25.436
81	0-9-83-76-86-65-55-64-102-98-100	1000	92.136
82	0-9-83-76-86-65-55-64-106-98-100	980	98.835
